# Phospholipid:Diacylglycerol Acyltransferase1 Overexpression Delays Senescence and Enhances Post-heat and Cold Exposure Fitness

**DOI:** 10.3389/fpls.2020.611897

**Published:** 2020-12-14

**Authors:** Kamil Demski, Anna Łosiewska, Katarzyna Jasieniecka-Gazarkiewicz, Sylwia Klińska, Antoni Banaś

**Affiliations:** Intercollegiate Faculty of Biotechnology, University of Gdańsk and Medical University of Gdańsk, Gdańsk, Poland

**Keywords:** PDAT, *Arabidopsis thaliana*, plant stress, plant senescence, LPEAT, LPCAT, heat-shock, cold stress

## Abstract

In an alternative pathway to acyl-CoA: diacylglycerol acyltransferase (DGAT)-mediated triacylglycerol (TAG) synthesis from diacylglycerol, phospholipid:diacylglycerol acyltransferase (PDAT) utilizes not acyl-CoA but an acyl group from sn-2 position of a phospholipid, to form TAG. The enzyme’s activity *in vitro* matches DGAT’s in a number of plant species, however its main function in plants (especially in vegetative tissue) is debatable. In the presented study, we cultivated *PDAT1*-overexpressing, *pdat1* knockout and wild-type lines of *Arabidopsis thaliana* through their whole lifecycle. *PDAT1* overexpression prolonged Arabidopsis lifespan in comparison to wild-type plants, whereas knocking out *pdat1* accelerated the plant’s senescence. After subjecting the 3-week old seedlings of the studied lines (grown *in vitro*) to 2-h heat stress (40°C) and then growing them for one more week in standard conditions, the difference in weight between wild-type and *PDAT*1-overexpressing lines increased in comparison to the difference between plants grown only in optimal conditions. In another experiment all lines exposed to 2-week cold stress experienced loss of pigment, except for *PDAT*1-overexpressing lines, which green rosettes additionally weighed 4 times more than wild-type. Our results indicate that plants depleted of *PDAT1* are more susceptible to cold exposure, while *PDAT1* overexpression grants plants a certain heat and cold resilience. Since it was shown, that lysophospholipids may be intertwined with stress response, we decided to also conduct *in vitro* assays of acyl-CoA:lysophosphatidylcholine acyltransferase (LPCAT) and acylCoA:lysophosphatidylethanolamine acyltransferase (LPEAT) activity in microsomal fractions from the *PDAT1*-overexpressing Arabidopsis lines in standard conditions. The results show significant increase in LPEAT and LPCAT activity in comparison to wild-type plants. *PDAT1*-overexpressing lines’ rosettes also present twice as high expression of *LPCAT2* in comparison to control. The presented study shows how much heightened expression of *PDAT1* augments plant condition after stress and extends its lifespan.

## Introduction

Triacylglycerols (TAG) are a plant’s way of storing high-dense energy, being twice as energy-efficient as equivalent mass of carbohydrates or protein (Xu and Shanklin, 2016). Those most-abundant neutral lipids are found in almost all plant tissues and have multiple functions in plants (Stumpf and Conn, 1988; Kaup et al., 2002; Kim et al., 2002; Theodoulou and Eastmond, 2012; Fan et al., 2013a,b). Recently, stress conditions were shown to promote TAG synthesis in plants by providing fatty acids from autophagy of cellular organelles (Su et al., 2020).

Triacylglycerol had been considered to be only synthesized in Kennedy pathway from acyl-CoA and diacylglycerol (DAG) by DGAT enzyme (acyl-CoA:diacylglycerol acyltransferase; Kennedy, 1961). However, in the year 2000 an enzyme was discovered, which was able to form TAG not by utilizing acyl-CoA, but by taking an acyl group from a phospholipid (most of the time phosphatidylcholine or phosphatidylethanolamine) and transferring it to the sn-3 position of DAG. This enzyme has been known ever since as phospholipid:diacylglycerol acyltransferase (PDAT; Banaś et al., 2000; Dahlqvist et al., 2000).

For the last 20 years, since its discovery, PDAT has been studied to a certain extent. The enzyme’s expression was confirmed in a number of plant species, including model organism *Arabidopsis thaliana*, *Vernonia galamensis*, *Euphorbia lagascae*, *Stokesia levis*, *Ricinus communis*, *Glycine max* and *Linum usitatissimum* among others (Ståhl et al., 2004; Mhaske et al., 2005; Li et al., 2010; Pan et al., 2013).

In plants, the exact contribution to TAG synthesis of PDAT-type enzymes in relation to DGAT enzymes remains elusive. PDAT role might vary depending on the plant species studied. In seeds of sunflower (*Helianthus annuus*), for example, PDAT’s role in TAG formation is dwarfed by DGAT’s role in the same process. The opposite is true for safflower (*Carthamus tinctorius*) in which seeds PDAT’s part in TAG accumulation matches, or even borders on surpassing DGAT’s part (Banaś et al., 2013).

Complementary role of DGAT and PDAT enzymes was also researched in Arabidopsis. In *A. thaliana pdat1* knockout lines *DGAT1* expression increases. Contrary, in Arabidopsis *dgat1* knockout lines, PDAT1 takes over as the primary contributor to TAG synthesis. Double mutants, depleted of both *dgat1* and *pdat1*, are not viable (Zhang et al., 2009).

The studies of PDAT’s activity illustrated that the enzyme may play a crucial role in plants, especially in those accumulating polyunsaturated or unusual fatty acids (Brown et al., 2012). Arabidopsis overexpressing *PDAT1* and *PDAT2* from linseed was enriched in PUFA (Pan et al., 2013). In *Ricinus communis* (producing hydroxy ricinoleic acid) or in *Crepis palaestina* (producing epoxy vernolic acid) PDAT preferentially utilized phospholipid’s ricinoleoyl or vernoyl groups to acylate sn-3 position of DAG (Dahlqvist et al., 2000). The transfer of oxygenated fatty acids directly from a membrane-bound phospholipid and not from the acyl-CoA pool could be connected with plant’s stress response (Mhaske et al., 2005).

Phospholipid:diacylglycerol acyltransferase enzymes are grouped into two families of PDAT1 and PDAT2 enzymes (Ståhl et al., 2004). The number of isoforms of each PDAT and their expression patterns differ between plant species (Yuan et al., 2017a; Chellamuthu et al., 2019). Moreover, different *PDAT* isoforms’ expression is upregulated during different stress conditions (Yuan et al., 2017a).

Phospholipid:diacylglycerol acyltransferase’s role during plant stress response seems to be significant. In *Camelina sativa PDAT*’s expression grew 2 to 5-fold during various stress conditions (Yuan et al., 2017b). Also, PDAT was shown to be crucial to plant basal thermotolerance through TAG synthesis. Arabidopsis *pdat*-depleted mutants were not able to accumulate TAG after subjecting them to heat stress (Mueller et al., 2017).

In this study, we decided to investigate PDAT’s role in *Arabidopsis thaliana* further. Cultivation of the following Arabidopsis lines in pot cultures: *PDAT1*-overexpressing, *pdat1* knockout and wild-type lines resulted in the discovery of PDAT1’s delaying effect on plant’s senescence. We then decided to test our lines in stress conditions. Heat-shock experiments revealed that *PDAT1*’s expression increases in heat-stressed Arabidopsis, and high-temperature treatment elevates the already increased weight of *PDAT1*-overexpressing plants. While testing cold stress we found loss of pigment in wild-type and knockout lines (which were still smaller than wild-type), but *PDAT1*-overexpressing lines retained chlorophyll in most plants and its rosette leaves were quadruple the weight of wild-type ones. We concluded that *PDAT1* overexpression equips Arabidopsis with a measurable heat and cold resilience. *In vitro* assays of acyl-CoA:lysophosphatidylcholine acyltransferase (LPCAT) and acyl-CoA:lysophosphatidylethanolamine acyltransferase (LPEAT) activity in microsomal fractions from the *PDAT1*-overexpressing Arabidopsis were also conducted with results showing a significant increase in LPLAT activity in comparison to wild-type plants, with an additional doubling of *LPCAT*2 expression detected in *PDAT1*-overexpressing plants. The implications of our findings on better understanding PDAT1’s role in plant stress mechanism are expanded on in the Discussion.

## Materials and Methods

### Plant Material

Our wild-type/control Arabidopsis was *Arabidopsis thaliana* ecotype Columbia-0 (Col-0). Knockout mutants of *pdat1* are homozygotes of T-DNA insertion mutant lines SALK_065334 (*pdat1* KO1) and SALK_032261 (*pdat1* KO2), which were obtained from the Arabidopsis Biological Resource Center (The Ohio State University). PCR screening for *pdat1* homozygotes was performed using primers detailed in Supplementary Table 1. Genomic DNA for the PCR was isolated from 4-week old leaves by phenol extraction method (Sambrook et al., 1989) and the reaction was performed using Taq DNA Polymerase (Thermo Fisher Scientific).

*AtPDAT1* (At5g13640) overexpression lines were kindly provided by Anders S. Carlsson in pART27 vector, with *AtPDAT1* being expressed under the 35S Cauliflower Mosaic Virus promoter (CaMV; Ståhl et al., 2004; Banaś et al., 2014).

### Plant Lifecycle Analysis

Lifecycle events of *pdat1* knockout mutant, *PDAT1*-overexpressing, and wild-type lines were recorded simultaneously during plant development. All of the studied lines were grown together in a growth chamber at 23°C. The applied photoperiod consisted of 16 h of light (120 μmol photons m^–2^ s^–1^) and 8 h of dark. The relative humidity was at 60%. Lifecycle events were recorded on 6 or more different plants for each line.

### *In vitro* Plant Cultivation

The seeds of tested Arabidopsis lines were surface-sterilized by immersion in ethanol (70%, 2 min) followed by 1 min wash in distilled water and immersion in calcium hypochlorite (4%, 10 min). After that, the seeds were washed with distilled water (4 times) and planted on plates containing: 1% agar, 0.33× Murashige-Skoog medium (MS) and 1% sucrose. The seeds were sown on agar on a straight line in an upper part of the plate (up to 10 seeds per plate) and put in 4°C for 48 h vernalization. After that the plates were placed in the growth chamber vertically. After the needed amount of time (depending on the experiment) the rosette leaves and roots were separated and weighed. Under all temperature conditions the plants were grown with 16 h light (120 μmol photons m^–2^ s^–1^)/8 h dark photoperiod. For optimal growth condition measurements, the plates were placed for 4 weeks in 23°C. For heat stress measurements, the plates where placed for 3 weeks in 23°C, then for 2 h in 40°C (in MLR-352H Climate Chamber, Panasonic; humidity set at 60%). Now, depending on the experimental destination they were either placed for 2 h back into 23°C (for relative expression measurements) or placed for an additional week back into 23°C (for fresh weight measurements, lipid extraction). For cold stress measurements, the plants were put into growth chamber with 23°C for 2 weeks and then for additional 2 weeks into cold chamber (4°C), after which the fresh weight was measured.

### Lipid Extraction and Analysis

Three 4-week-old rosettes of *in vitro* cultivated Arabidopsis were weighed and subjected to a Bligh and Dyer lipid extraction (Bligh and Dyer, 1959). One-fifth of the chloroform extract was transmethylated in 2 ml solution of 2% H_2_SO_4_ dissolved in dry methanol (45 min, 90°C), extracted to heptane with 50 nmol of heptadecanoic acid (internal standard) and then directly analyzed with GC-FID (Shimadzu GC-2010) utilizing 60 m × 0,25 mm BPX70 column (SGE Analytical Science). The rest of the chloroform extract was separated on TLC plate (silica gel 60; Merck) in heptane:diethyl ether:acetic acid (70:30:1) mobile phase. TAG spots were visualized under UV light after staining by 0.05% primuline solution and later scraped off. Silica gel samples containing TAG lipid group were from then on processed as described above for the one-fifth of the chloroform extract.

### Microsomal Fraction Isolation

To obtain microsomal fraction from roots and rosettes, the seeds of tested Arabidopsis lines were sterilized and placed on MS plates as described above (only spread throughout the plate and not in a straight line). After 2 weeks of cultivation in 23°C, they were transferred into flasks containing 0.5× MS medium and 1% sucrose and grown with gentle shaking (80 rpm) for three more weeks. For all 5 weeks *in vitro* Arabidopsis lines were cultivated under the same light conditions as pot-cultivated plants (16 h light/8 h dark). The roots from approximately 20 plants were then carefully separated from leaves and homogenized separately in a glass homogenizers with homogenization buffer (0.1 M potassium phosphate buffer with 7.2 pH, 0.33 M sucrose, 1 mg/ml BSA and catalase 1000 units/ml) in a cold room (4°C) in ice. After homogenates were ready they were filtered through two-layer Miracloth and centrifuged at 20 000 × *g* for 12 min. The supernatants were filtered through two-layer Miracloth and centrifuged again, this time at 100 000 × *g* for 100 min. The supernatants were disposed of and the obtained pellets (containing microsomal fraction) were suspended in 0.1 M potassium phosphate buffer (pH 7.2). Aliquots of each suspension were used to measure microsomal protein content via Pierce^TM^ BCA Protein Assay Kit (Thermo Fisher Scientific) and analyze phosphatidylcholine (PC) content as previously described by Klińska et al. (2019). PC content is equivalent to membrane concentrations in microsomal fractions. We show how both values relate to each other in Supplementary Table 4.

### Enzyme Assays

For conducting enzyme assays we have utilized substrates both employed by the enzymes and with proven *in vitro* activity (Ståhl et al., 2004; Banaś et al., 2014; Jasieniecka-Gazarkiewicz et al., 2017). For LPCAT activity tests, following optimization, 5 nmol of exogenous sn-1-18:1-lysophosphatidylcholine and 5 nmol of exogenous [^14^C]18:2-CoA were used with aliquots of microsomal fractions equivalent to 0.05 nmol (both roots and rosettes) of endogenous PC (0.022 μg of microsomal protein). The reaction components were diluted in 100 μl of 100 mM potassium buffer (pH 7.2). Addition of microsomal fraction was treated as the beginning of the reaction, which lasted for 30 min, and was conducted in 30°C. The reaction was terminated by addition of 375 μl of chloroform:methanol (1:2; v:v), 5 μl of glacial acetic acid, 125 μl of chloroform and 125 μl of water. Chloroform fraction was separated by centrifugation and transferred to a new tube. Extracts were then separated on silica gel 60 plates (Merck) utilizing thin-layer chromatography (TLC) principles in a glass chamber containing chloroform:methanol:acetic acid:water (90:15:10:2,5; v:v:v:v). The product of the reaction ([^14^C]-PC was visualized and quantified with electronic autoradiography (Instant Imager, Packard Instrument Co.).

AcylCoA:lysophosphatidylethanolamine acyltransferase activity tests were conducted and assessed in similar fashion, only instead of sn-1-18:1-lysophosphatidylcholine, sn-1-18:1-lysophosphatidylethanolamine was used, and microsomal fraction concentration went up to 0.5 nmol (0.22 μg of microsomal protein for both roots and rosettes) of endogenous PC per assay. The desired product was not [^14^C]-PC, but [^14^C]-PE.

Phospholipid:diacylglycerol acyltransferase activity assays were conducted in a slightly different manner. 18 h before the planned activity assays aliquots of 12 nmol of microsomal fractions were lyophilized (Heto PowerDry LL3000 Freeze Dryer, Thermo Electron Corporation). 5 nmol of sn-1-18:1-sn-2-[^14^C]18:2-PE and 5 nmol of sn-1,2-18:1-diacylglycerol for each assay were diluted in benzene and added to lyophilized microsomal fraction. The benzene was then evaporated under nitrogen, and 100 μl of 100 mM potassium buffer (pH 7.2) was added (beginning of the reaction). The reaction lasted for 2 h in 30°C. The reaction was terminated as described above for LPCAT activity. Reagent-containing chloroform extracts were separated by TLC on silica gel 60 plates with solvent system of hexane:diethyl ether:acetic acid (70:30:1; v:v:v:v). The product, which was [^14^C]-TAG, was visualized and quantified with electronic autoradiography.

### Relative Expression Analysis

Before the experiments, primers for qPCR were designed (Supplementary Table 2) for the following genes: *ACT2* (At3g18780), *PP2A* (At1g69960), *LPCAT1* (At1g12640), *LPCAT2* (At1g63050), *LPEAT1* (At1g80950), *LPEAT2* (At2g45670), *PDAT1* (At5g13640), *ATG8a* (At4g16520). Heat-stressed or non-stressed leaves of 3-week-old Arabidopsis grown *in vitro* were flash-frozen in liquid nitrogen. Total RNA was extracted from samples with GeneMatrix Universal RNA Purification Kit (EurX). RNA was then incubated with dsDNase (Thermo Fisher Scientific) to remove genomic DNA and then cDNA was synthesized with Maxima First Strand cDNA Synthesis Kit for RT-qPCR (Thermo Fisher Scientific). Maxima SYBR Green/ROX qPCR Master Mix (2×; Thermo Fisher Scientific) was used for qPCR analysis. qPCR measurements were conducted in QuantStudio^TM^ 3 Real-Time PCR System (Applied Biosystems). All procedures were performed according to the manufacturers’ instructions. The acquired results were analyzed employing the 2^–ΔΔ*CT*^ algorithm (Winer et al., 1999).

## Results

### *AtPDAT1* Overexpression Prolongs *A. thaliana* Flowering and Delays Senescence

**FIGURE 1:**
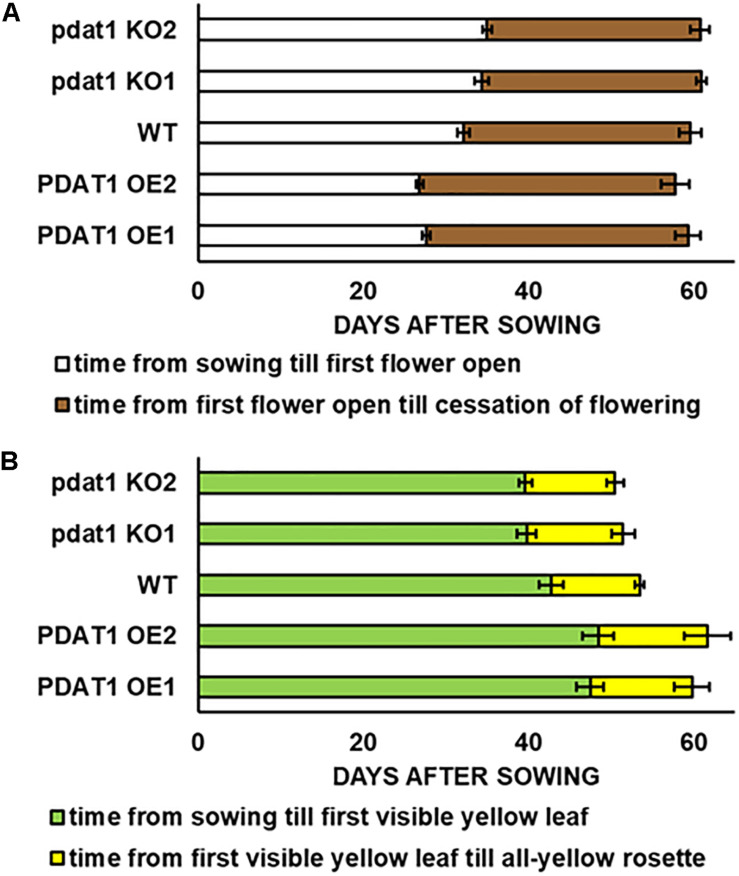
Differences in length of *Arabidopsis thaliana* lifecycle stages: time of flowering **(A)** and rosette senescence **(B)**, measured in days after sowing between wild-type (WT), *PDAT1*-overexpressing lines (PDAT1 OE1 and OE2) and *pdat1* knockout lines (pdat1 KO1 and KO2). Error bars indicate standard deviations (SD) between biological replicates (*n* = 6).

We began our journey into investigating *At*PDAT1 by studying phenotypic differences between selected *pdat1* knockout mutants and *PDAT1*-overexpressing lines, as well as wild-type *Arabidopsis thaliana*. After 48 h vernalization, the seeds were planted into soil and observed while growing under assigned standard conditions (23°C, 16 h light/8 h dark photoperiod, 60% humidity). Key plant lifecycle events which were examined under utmost scrutiny were: appearance of the first flower buds (known as 0 DAF – day after flowering), opening of the first flower, yellowing of the first rosette leaf, yellowing of the last leaf in the rosette and cessation of flowering (all flowers of the plant becoming siliques). The obtained results are presented in Figure 1 and Supplementary Table 3.

**FIGURE 2:**
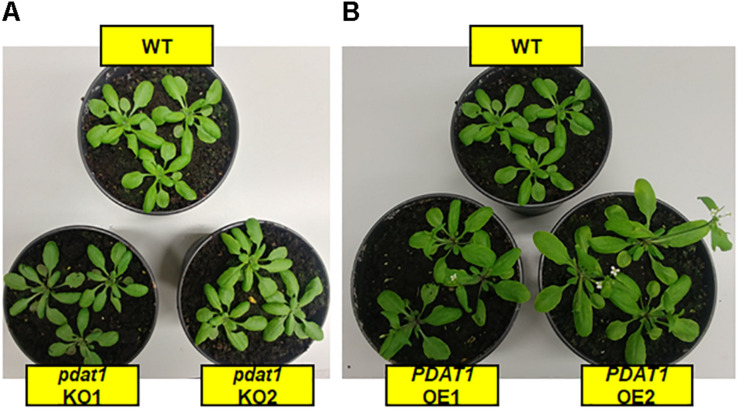
Cultivation of studied *Arabidopsis thaliana* lines in pots, 4 weeks after sowing. Panel **(A)** shows *pdat1* knockout lines in comparison to wild-type control, while panel **(B)** shows the same wild-type control with *PDAT1*-overexpressing lines.

Contrary to our expectations, there was no drastic difference between wild-type and *pdat1* knockout lines generative lifecycle events. Wild-type and mutant lines went through the main milestones in flower development at similar points in time (they occurred at similar days after sowing), from first appearance of flower buds, through first flower opening to cessation of flowering, understood as all flowers on a single plant becoming siliques. *PDAT1*-overexpressing lines, on the other hand, began flowering significantly earlier than control, but ceased to produce flowers around the same time as wild-type and mutant lines. Their generative lifecycle was thus effectively longer. The wild-type plants were also visibly smaller than *PDAT1*-overexpressing plants (Figure 2).

Both the knocking-out and overexpression of *PDAT1*, influenced the advancement of Arabidopsis leaves yellowing (Figure 1). Lines with knockout of *pdat1*, entered senescence earlier than wild-type and had an all-yellow leaves rosette sooner. Overexpression of the gene, on the other hand, retarded plant aging and resulted in *PDAT1*-overexpressing plants going through the appearance of the first yellow leaf in the rosette at average 5 or 6 days later than wild-type (depending on the *PDAT1*-overexpressing line) and exhibiting an all-yellow leaves rosette 6 or 8 days after wild-type, on average. Therefore, *PDAT1* expression level acts on Arabidopsis senescence and lifespan.

### Overexpression and Knockout of *AtPDAT1* Influence Plant Rosette and Root Weight *in vitro*

**FIGURE 3:**
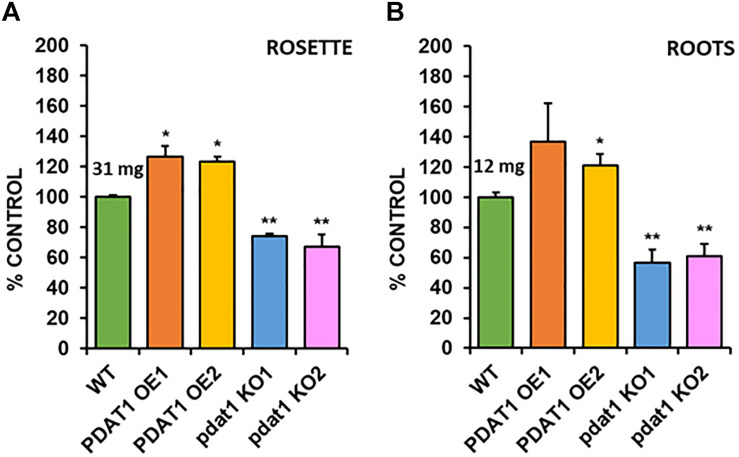
Differences in rosette **(A)** and root **(B)** fresh weight of *Arabidopsis thaliana* seedlings between wild-type (WT, treated as control), *PDAT1*-overexpressing lines (PDAT1 OE1 and OE2) and *pdat1* knockout lines (pdat1 KO1 and KO2). The measurements were taken 4 weeks after planting seeds *in vitro*. Mean weight of each control is written above the bar representing it. Error bars indicate standard deviations (SD) between 3 average weights of 3 groups of 10 pooled seedlings (*n* = 3). Single asterisks (*) indicate significant difference between means in comparison to control (WT) in a two-tailed Student’s *t*-test at *p* < 0.05 and double asterisks (**) at *p* < 0.01.

In order to check the effect, which both *PDAT1* overexpression and *pdat1* knockout would have on plant size (weight), we cultivated *A. thaliana* lines *in vitro* in assigned standard conditions, described in the previous chapter. Subsequently, we measured the fresh weight of their rosettes and roots after 4 weeks had passed (Supplementary Figure 1 contains photos of the cultivation at 3 weeks). The rosettes of *PDAT1*-overexpressing Arabidopsis lines weighed significantly more than wild-type control (126 and 123% of the control weight; Figure 3A). Clear difference was also observed in *pdat1* mutants, which weighed 67 and 74% of control weight, depending on the mutant line. The differences were also significant in roots (Figure 3B), especially between *pdat1* knockout plants and control, since the *pdat1* mutant Arabidopsis had significantly lower root weight at 56 and 61% of the weight of control’s roots. No trends in regard to total fatty acid composition were observed (Supplementary Figure 2). In TAG there was a slight decrease in molar proportion of 18:3 in *pdat1* knockout lines (Supplementary Figure 3), which would be in accordance with PDAT1’s specificity toward unsaturated fatty acids.

### *AtPDAT1* Influences Arabidopsis Fitness When Subjected to Stress Factors

**FIGURE 4:**
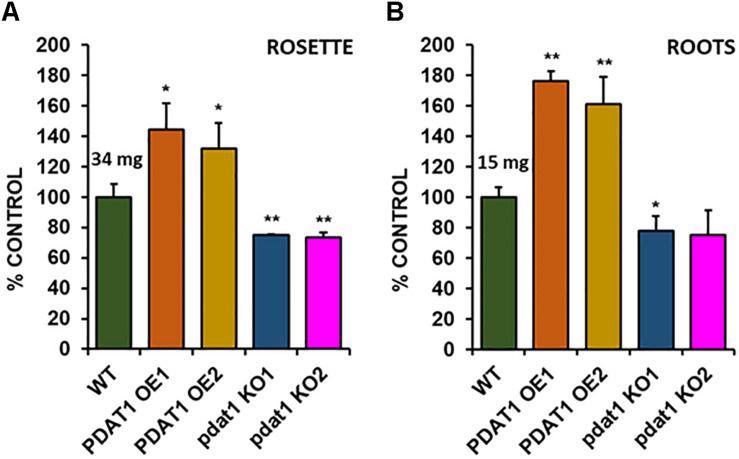
Comparison of wild-type (WT), *PDAT1*-overexpressing (PDAT1 OE1 and OE2) and *pdat1* knockout lines (pdat1 KO1 and KO2), when exposed to 2 h 40°C heat stress at 3-week-old and then placed for one more week in standard conditions. Fresh weight of either rosettes **(A)** or roots **(B)** is compared. Mean weight of each control (WT) is written above the bar representing it. Error bars indicate standard deviations (SD) between 3 average weights of 3 groups of 10 pooled seedlings (*n* = 3). Single asterisks (*) indicate significant difference between means in comparison to control (WT) in a two-tailed Student’s *t*-test at *p* < 0.05 and double asterisks (**) at *p* < 0.01.

Because of the previous experiment’s outcome we decided to test the investigated Arabidopsis lines in stress conditions, to see, if *PDAT1* overexpression or knockout were influencing plant’s resilience to hostile environmental conditions. The first series of experiments we designed focused on short-term heat stress. In our experiment 3-week-old Arabidopsis seedlings grown *in vitro* in optimal temperature (23°C) were exposed to heat for 2 h (40°C, 60% humidity). After the shock treatment, plants were placed back into optimal temperature, where they recovered for 7 days (Supplementary Figure 4). Then we proceeded to measure the fresh weight of all investigated plants’ rosettes (Figure 4A) and roots (Figure 4B).

**FIGURE 5:**
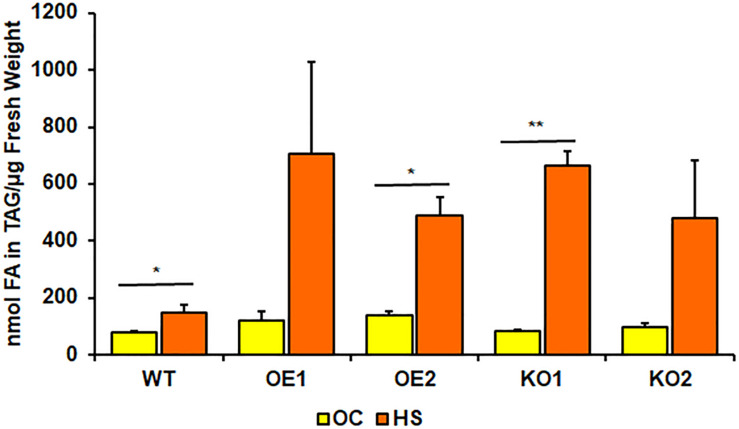
Comparison of triacylglycerol (TAG) content in rosettes of wild-type (WT), *PDAT1*-overexpressing lines (OE1 and OE2) and *pdat1* knockout lines (KO1 and KO2), measured as nmol of all fatty acids in the TAG fraction per μg of fresh weight. The comparison was made between 4-week-old Arabidopsis seedlings grown only in optimal conditions (OC) and seedlings which were subjected to 2-h heat-shock at 40°C 3 weeks after sowing (HS). Error bars indicate standard deviations (SD) between 3 means of biological replicates (*n* = 3). Asterisks indicate significant difference in TAG content between means of the same line in optimal conditions (OC) and after heat stress (HS) in a two-tailed Student’s *t*-test. Single asterisks (*) indicate significant difference at *p* < 0.05 and double asterisks (**) at *p* < 0.01.

Rosettes of wild-type (Figure 4A) experienced a small boost in comparison with 4-week-old seedlings, grown only in optimal temperature (Figure 3A). Heat-shocked knockout lines’ rosettes also weighed more than those grown in optimal temperature, but their weight in relation to the stressed control mirrored optimal conditions. However, that was not true for root measurements (Figure 4B). The weight of *pdat1* mutant roots constituted more percentage of the control than their unstressed counterparts in previous experiment – 78 and 75% of control’s root weight in comparison to 56 and 61% in optimal conditions. The biggest change comparing to unstressed Arabidopsis *in vitro* growth occurred in *PDAT1*-overexpressing plants’ roots. The difference between them and control grew 40% in case of both overexpressing lines in comparison to optimal conditions. The weight divergence between wild-type and PDAT1-overexpressing plants increased also in case of rosettes (by 18% and 9% for particular lines). Total fatty acid composition was not affected by changes observed in the studied lines (Supplementary Figure 5). What distinguished the heat-shocked plants from the non-stressed ones was the TAG content, which increased in all of the studied lines (Figure 5). However, we could not discern any trends between the wild-type, the overexpressing lines and the mutant lines. Similarly, to non-stressed Arabidopsis, TAG of knockout lines contained lower mole percent of 18:3 in comparison to other studied plants (Supplementary Figure 6).

**FIGURE 6:**
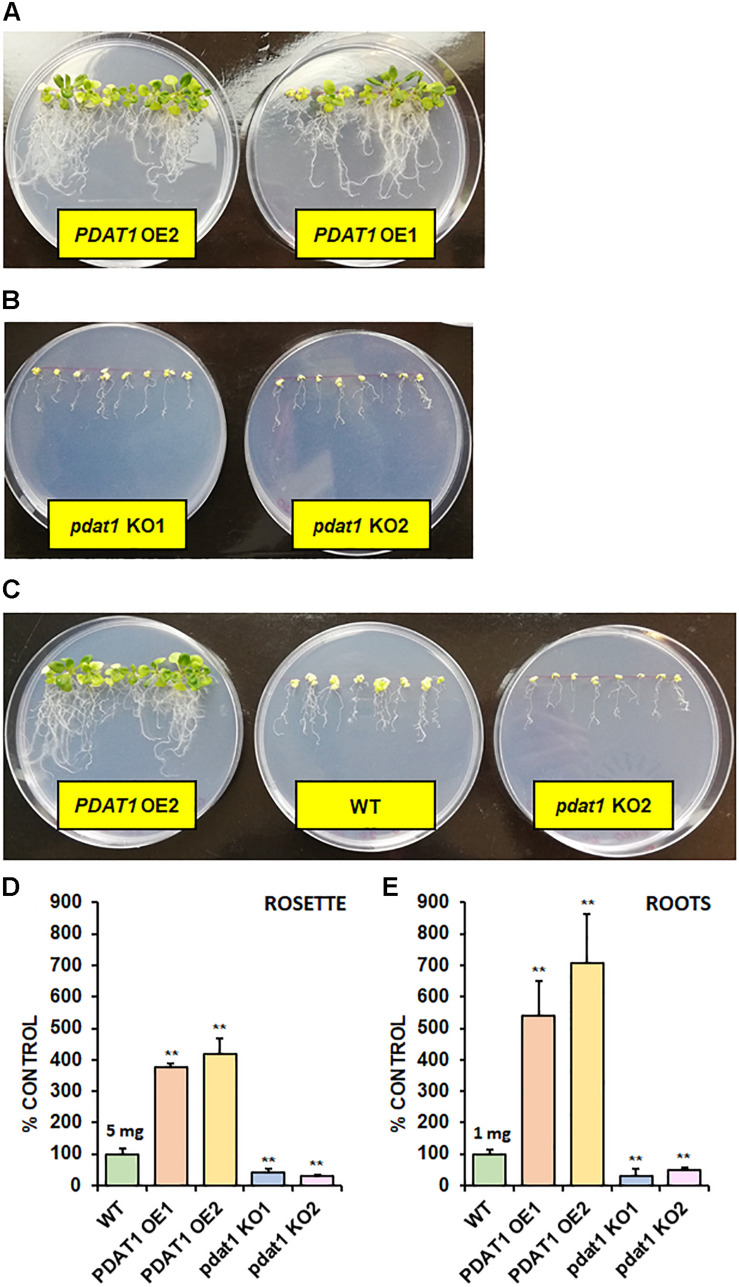
The effect of 2-week 4°C cold stress on 4-week-old seedlings of *Arabidopsis thaliana* lines cultivated *in vitro*. Panel **(A)** presents two *PDAT1*-overexpressing lines, panel **(B)** presents two *pdat1* knockout lines, and panel **(C)** presents, from left to right: *PDAT1*-overexpressing line (*PDAT1* OE2), wild-type control (WT) and *pdat1* knockout line (*pdat1* KO2). Diameter of the Petri dishes used is 90 mm. Fresh weight of the cold-subjugated Arabidopsis lines was in both rosettes **(D)** and roots **(E)**. Mean weight of each control (WT) is written above the bar representing it. Error bars indicate standard deviations (SD) between 3 average weights of 3 groups of 10 pooled seedlings (*n* = 3). Single asterisks (*) indicate significant difference between means in comparison to control (WT) in a two-tailed Student’s *t*-test at *p* < 0.05 and double asterisks (**) at *p* < 0.01.

After short-term heat stress, we decided to subject our Arabidopsis lines to prolonged cold exposure. We selected 4°C as our testing cold temperature. Knowing, the plants would not germinate in 4°C, we first cultivated them in optimal temperature for 2 weeks (*in vitro*) and then placed them in the cold chamber for the next 2 weeks. Both wild-type control and *pdat1* knockout reacted extremely to the stress factor, experiencing arrested growth and loss of pigment (Figures 6A–C). *PDAT1*-overexpressing lines were the most resilient, continuing to grow, with most or all of the rosette leaves preserving the green color. The measured fresh weight of rosette leaves and roots reflected that (Figures 6D,E). While both rosettes and roots of the *pdat1* mutant lines weighed significantly less than control’s rosettes and roots, the *PDAT1*-overexpressing lines’ rosettes weighed on average 3.75 and 4.2 times more and roots average weight was 5.41 and 7.06 times more than their wild-type counterparts.

### Heat Stress Stimulates *PDAT1* Expression in Arabidopsis

Some of the Arabidopsis plants, which were subjected to short-term heat stress were not given 1 week to recover, but their rosettes were instead harvested 2 h after the high-temperature treatment and used for relative expression measurements. For comparison, relative expression was also measured in rosettes of 3-week-old seedlings, which were grown with heat-stressed plants, but not exposed to 2 h in 40°C.

**FIGURE 7:**
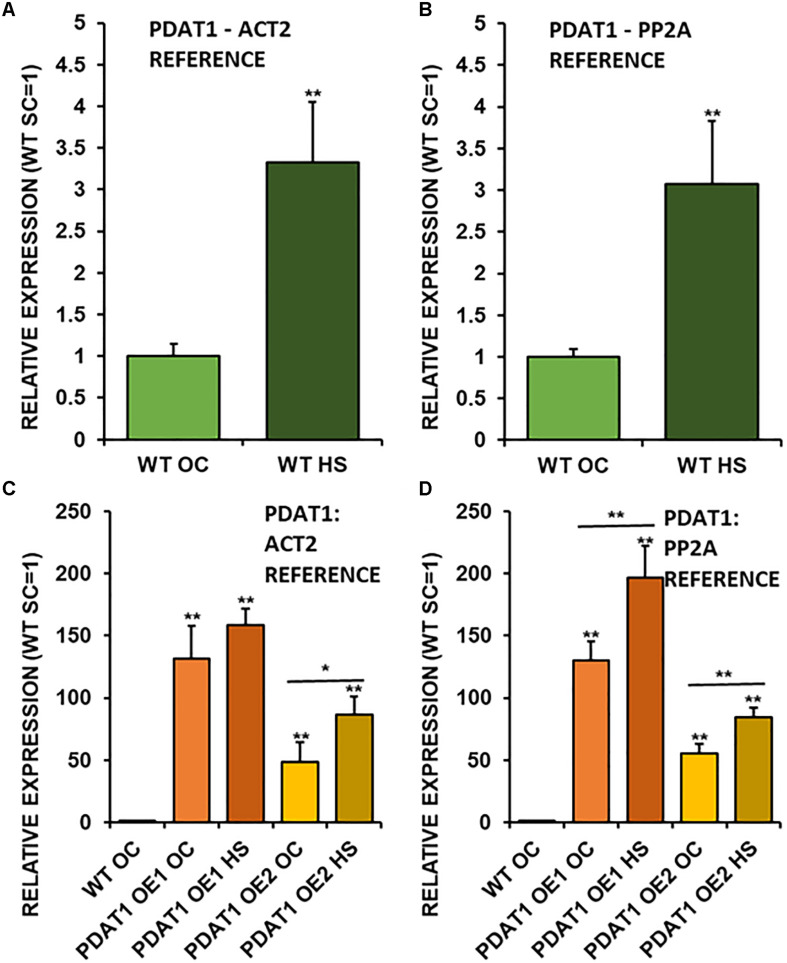
Relative expression of *AtPDAT1* in 3-week old rosettes of *Arabidopsis thaliana* in comparison to *ACT2* housekeeping gene **(A,C)** and *PP2A* housekeeping gene **(B,D)** as determined by RT-qPCR. First two charts **(A,B)** present difference in *AtPDAT1* expression between wild-type (WT) in optimal conditions (OC) and wild-type after heat stress (HS). Charts **(C,D)** present how relative *AtPDAT1* expression in wild-type in optimal conditions (WT OC) compares to *AtPDAT1* relative expression in *AtPDAT1*-overexpressing lines in optimal conditions (PDAT1 OE1 SC, PDAT1 OE2 SC) and *AtPDAT1*-overexpressing lines after heat stress (PDAT1 OE1 HS, PDAT1 OE2 HS). Error bars indicate standard deviations (SD) between biological replicates (*n* = 3). Double asterisks (**) above error bars indicate significant difference between means in comparison to control (WT OC) in a two-tailed Student’s *t*-test at *p* < 0.01. Single asterisks (*) or double asterisks (**) above horizontal lines above two columns representing the same Arabidopsis line subjected to different growth conditions indicate significant difference between means of the results in in a two-tailed Student’s *t*-test at *p* < 0.05 and *p* < 0.01, respectively.

Taking into the account just wild-type lines, it turns out, that *PDAT1* is expressed more than three times more in rosettes of seedlings, which were cultivated in 40°C for 2 h (Figures 7A,B) in comparison to non-stressed wild-type. It means, that enduring a short period of heat exposure triggers tripling of *PDAT1* expression in Arabidopsis plants.

Investigated *PDAT1*-overexpressing lines exhibited a monumental increase in *PDAT1* relative expression in comparison to wild-type (Figures 7C,D), just in optimal conditions. The increase of *PDAT1* expression between *PDAT1*-overexpressing lines in optimal conditions versus the same lines after heat stress was noticeable and statistically significant in one *PDAT1*-overexpressing line in reference to *ACT2* and in both lines in reference to *PP2A* (Figures 7A,B).

### *ATG8a* Post-heat-Stress Expression Is Not Conditional on *PDAT1* Expression

Since plant stress response stimulates autophagocytic process, we decided to measure relative expression levels of *ATG8a* gene, which encodes a protein regarded as essential in autophagy (Bu et al., 2020). *ATG8*a expression levels were measured in wild-type plants, *PDAT1*-overexpressing plants and pdat1 knockout mutants, both those cultivated only in optimal temperature for 3 weeks and those exposed to 2 h heat stress with 2 additional hours after shock treatment.

**FIGURE 8:**
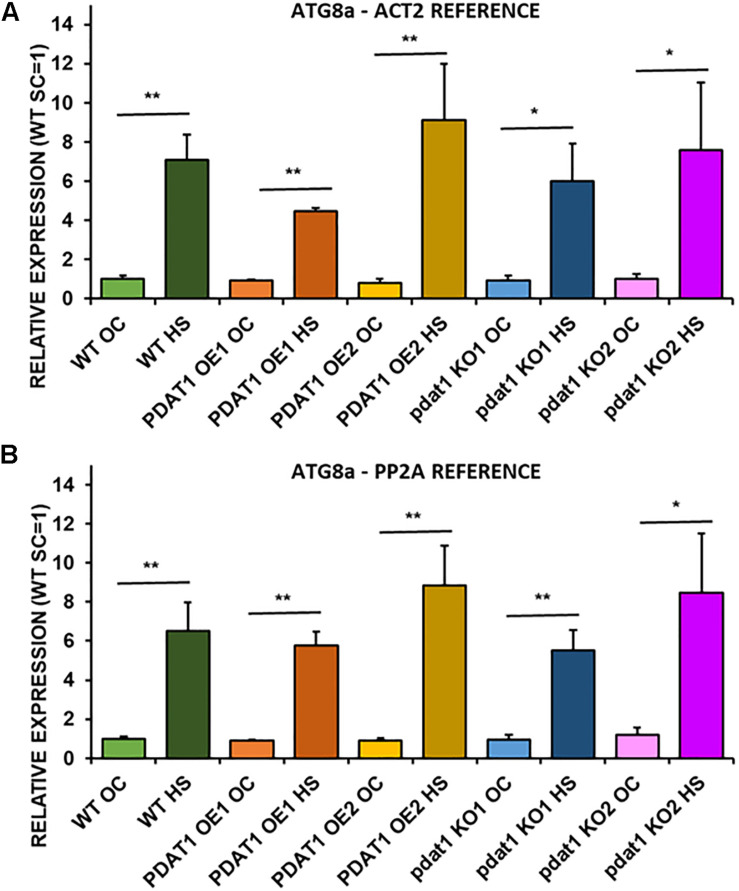
Relative expression of *ATG8a* in 3-week old rosettes of *Arabidopsis thaliana* in comparison to *ACT2* housekeeping gene **(A)** and *PP2A* housekeeping gene **(B)** as determined by RT-qPCR. WT indicates wild-type line (control), PDAT1 OE1 indicates *AtPDAT1*- overexpressing line 1, PDAT1 OE2 indicates *AtPDAT1*- overexpressing line 2, pdat1 KO1 indicates line 1 with *pdat1* knockout and pdat1 KO2 indicates line 2 with *pdat1* knockout. OC refers to seedlings grown only in optimal conditions, whereas HS refers to seedlings, which were subjected to 2 h heat stress. Error bars indicate standard deviations (SD) between biological replicates (*n* = 3). Asterisks indicate significant difference in relative *ATG8a* expression between means of the same line in optimal conditions (OC) and after heat stress (HS) in a two-tailed Student’s *t*-test. Single asterisks (*) indicate significant difference at *p* < 0.05 and double asterisks (**) at *p* < 0.01.

*ATG8a* relative expression was on the same level and not statistically different in any of the lines cultivated without heat-shock treatment (Figure 8). In comparison, all *ATG8*a expression levels in every Arabidopsis line subjected to high temperature were significantly multiple times higher than optimal-temperature-only wild-type control. The highest *ATG8a* relative expression in heat-exposed plants was found in *PDAT1*-overexpressing line 2 followed by expression in *pdat1* knockout mutant line 2, then wild-type, then *pdat1* knockout line 1 with the lowest ATG8a expression after heat exposure presented by *PDAT1*-overexpressing line 1. Expression in all plants subjected to heat-shock was on similar level. Based on those results, it seems that *PDAT1* overexpression or knockout in *A. thaliana* does not correlate with higher or lower expression of autophagy-related *ATG8a* gene, neither in standard growth conditions nor after 2 h following the exposure to high-temperature stress.

### *AtPDAT1* Overexpression Lines Present Higher LPLAT Activity in Microsomal Fractions

**FIGURE 9:**
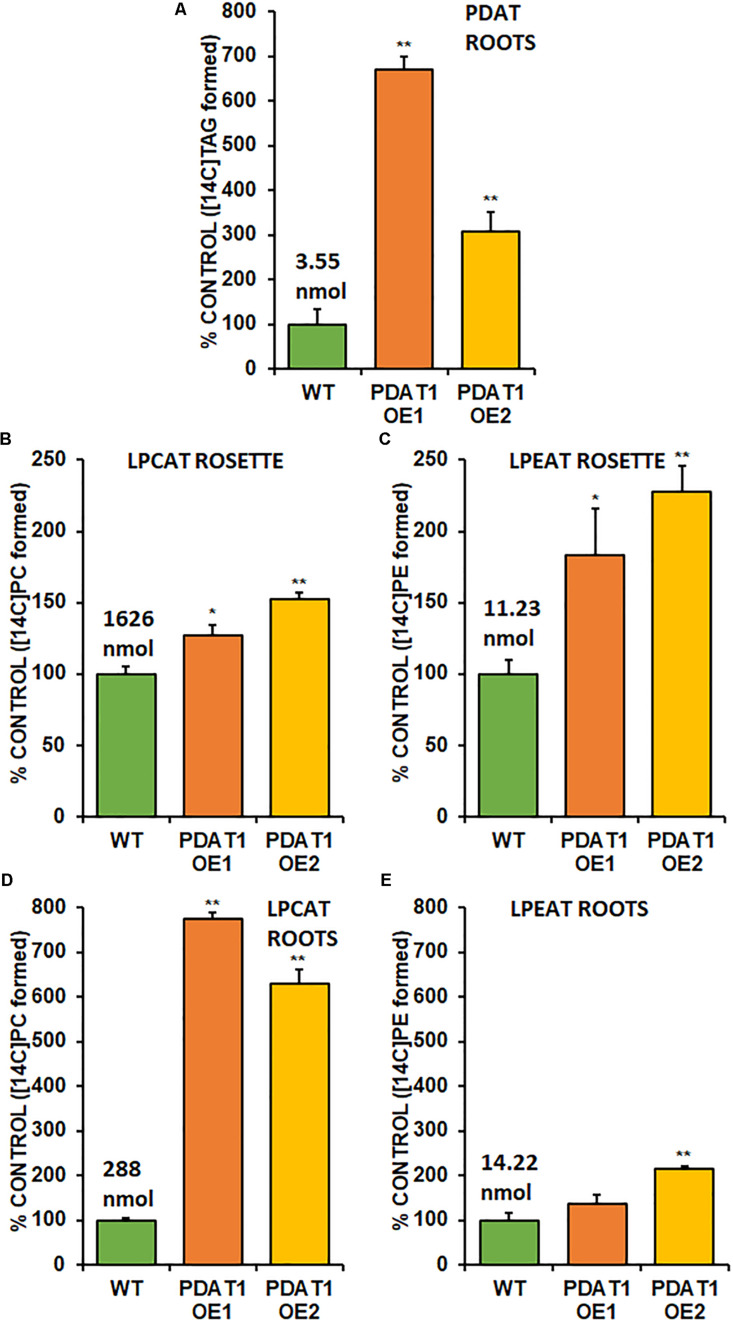
Endogenous enzyme activity measured *in vitro* in root **(A,B,C)** and rosette **(D,E)** microsomal fractions from Arabidopsis wild-type control (WT) and *PDAT1*-overexpressing lines (PDAT1 OE1 and PDAT1 OE2). Comparison of enzyme activity of PDAT **(A)**, LPCAT **(B,D)**, and LPEAT **(C,E)** between the two lines. Values above the WT bars correspond to 100% activity for each chart measured as nmol of [14C] enzymatic activity product (TAG for PDAT, PC for LPCAT and PE for LPEAT) synthesized during 1 h reaction, utilizing microsomal fraction equivalent to 1 mg of microsomal protein. Error bars indicate standard deviations (SD) between biological replicates (*n* = 3). Single (*) or double (**) asterisks indicate significant difference between means in comparison to control (WT) in a two-tailed Student’s *t*-test at *p* < 0.05 or at *p* < 0.01, respectively.

Phospholipid:diacylglycerol acyltransferase enzymes are intertwined with LPLAT enzymes, since products of forward reactions of LPLAT enzymes are substrates for PDAT in DAG acylation and one of the products of PDAT action (LPL) is a substrate of LPLAT enzymes. For that reason, we decided to conduct *in vitro* assays using microsomal fractions from rosettes and roots of wild-type and *PDAT1*-overexpressing Arabidopsis cultures. First, we measured PDAT endogenous activity in root microsomal fractions from both wild-type control and *PDAT1*-overexpressing lines to confirm that those lines showed higher enzyme activity *in vitro*. PDAT1 activity in roots (Figure 9A; Supplementary Figure 7A) correlated with *PDAT1* relative expression measurements in rosettes, even though plants for both experiments were grown in different conditions (Figures 7C,D). *PDAT1* overexpression in roots was confirmed in its endogenous enzyme activity, which was 6.7 and 3.1-fold higher in *PDAT1*-overexpressing lines than in wild-type control.

With higher activity in *PDAT1* OE confirmed, we advanced to LPLAT activity measurements. Since phosphatidylcholine (PC) and phosphatidylethanolamine (PE) are the most abundant phospholipids in plants, we concentrated on LPCAT and LPEAT activity of the microsomal fractions. We measured the analyzed enzyme’s activity as amount of product (an appropriate phospholipid) *de novo* synthesized in an *in vitro* reaction utilizing the amount of microsomal fraction containing 1 mg of microsomal protein during 1 h. Of the two LPLAT, LPCAT turned out to be the more active enzyme in microsomal fractions from both wild-type and *PDAT1*-overexpressing line (after optimizing reactions we used aliquots of root microsomal fraction equivalent to 0.05 nmol of endogenous PC for LPCAT assays and equivalent to 0.5 nmol of endogenous PC for LPEAT assays). LPCAT was significantly more active in *PDAT1*-overexpressing line 1 roots (7.7 times) and rosettes (by 27%) and *PDAT1*-overexpressing line 2 (6.3 times) roots and rosettes (by 52%) (Figures 9B,D; Supplementary Figures 7B,D). Our preliminary studies of microsomal LPLAT activity in wild-type and *PDAT1*-overexpressing line 2 (Supplementary Figure 8) showed higher LPCAT activity in rosettes of PDAT1-overexpressing line 2 and lower LPCAT activity in the same line in roots. However, LPCAT activity was always higher in comparison to wild-type control.

The investigated LPEAT activity was also higher in microsomal fraction from PDAT1-overexpressing lines in both rosettes and roots (Figures 9C,E; Supplementary Figures 7C,E). The increase in LPEAT activity in rosettes was more pronounced reaching twice the activity of control in both PDAT1-overexpressing lines.

### *AtLPCAT2* Expression Doubles in *AtPDAT1* Overexpression Lines

Knowing how *PDAT1* overexpression affects LPLAT activity *in vitro*, we proceeded to analyze the relative expression of two *LPCAT* and two *LPEAT* isoenzymes (*LPCAT1*, *LPCAT2*, *LPEAT1* and *LPEAT2*) in rosettes of 3-week-old plants cultivated in optimal growth conditions *in vitro*, as well as in rosettes of 3-week-old plants harvested after 2 h heat stress, followed by 2 h in optimal conditions.

**FIGURE 10:**
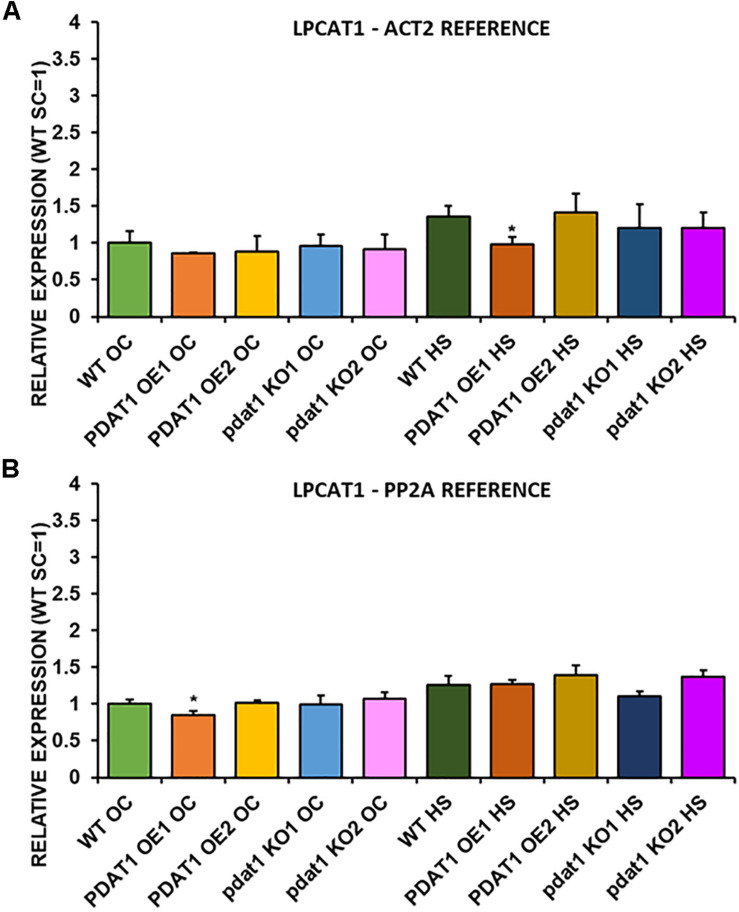
Relative expression of *LPCAT1* in 3-week old rosettes of *Arabidopsis thaliana* in comparison to *ACT2* housekeeping gene **(A)** and *PP2A* housekeeping gene **(B)** as determined by RT-qPCR. WT indicates wild-type line (control), PDAT1 OE1 indicates *AtPDAT1*- overexpressing line 1, PDAT1 OE2 indicates *AtPDAT1*- overexpressing line 2, pdat1 KO1 indicates line 1 with *pdat1* knockout and pdat1 KO2 indicates line 2 with *pdat1* knockout. OC refers to seedlings grown only in optimal conditions, whereas HS refers to seedlings, which were subjected to 2 h heat stress. Error bars indicate standard deviations (SD) between biological replicates (*n* = 3). Single asterisks (*) above error bars indicate significant difference at *p* < 0.05 in a two-tailed Student’s *t*-test between means of the tested line and the wild-type line grown in the same conditions (OC or HS).

**FIGURE 11:**
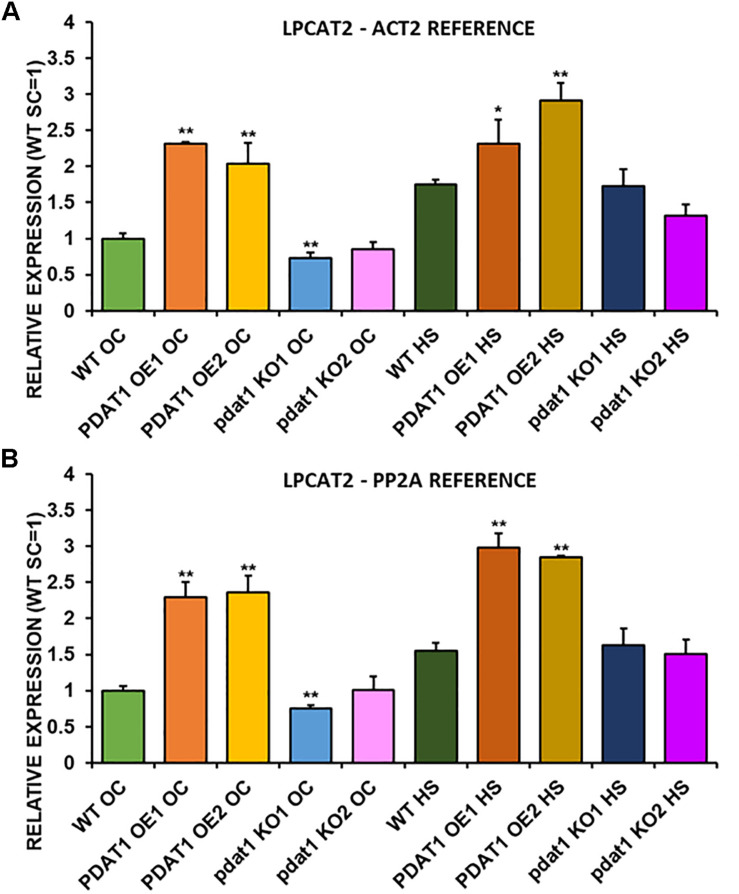
Relative expression of *LPCAT2* in 3-week old rosettes of *Arabidopsis thaliana* in comparison to *ACT2* housekeeping gene **(A)** and *PP2A* housekeeping gene **(B)** as determined by RT-qPCR. WT indicates wild-type line (control), PDAT1 OE1 indicates *AtPDAT1*- overexpressing line 1, PDAT1 OE2 indicates *AtPDAT1*- overexpressing line 2, pdat1 KO1 indicates line 1 with *pdat1* knockout and pdat1 KO2 indicates line 2 with *pdat1* knockout. OC refers to seedlings grown only in optimal conditions, whereas HS refers to seedlings, which were subjected to 2 h heat stress. Error bars indicate standard deviations (SD) between biological replicates (*n* = 3). Single (*) and double (**) asterisks above error bars indicate significant difference at *p* < 0.05 and at *p* < 0.01, respectively, in a two-tailed Student’s *t*-test between means of the tested line and the wild-type line grown in the same conditions (OC or HS).

**FIGURE 12:**
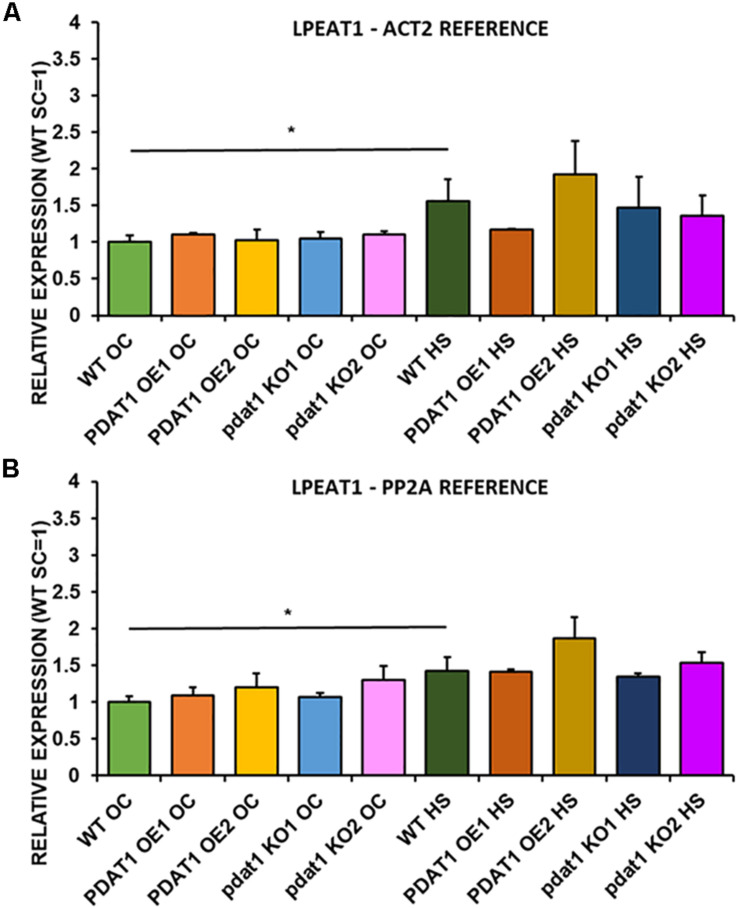
Relative expression of *LPEAT1* in 3-week old rosettes of *Arabidopsis thaliana* in comparison to *ACT2* housekeeping gene **(A)** and *PP2A* housekeeping gene **(B)** as determined by RT-qPCR. WT indicates wild-type line (control), PDAT1 OE1 indicates *AtPDAT1*- overexpressing line 1, PDAT1 OE2 indicates *AtPDAT1*- overexpressing line 2, pdat1 KO1 indicates line 1 with *pdat1* knockout and pdat1 KO2 indicates line 2 with *pdat1* knockout. OC refers to seedlings grown only in optimal conditions, whereas HS refers to seedlings, which were subjected to 2 h heat stress. Error bars indicate standard deviations (SD) between biological replicates (*n* = 3). Single (*) asterisks above horizontal lines indicate significant difference in a two-tailed Student’s *t*-test at *p* < 0.05 between means of the results two columns at the border of the horizontal line represent.

**FIGURE 13:**
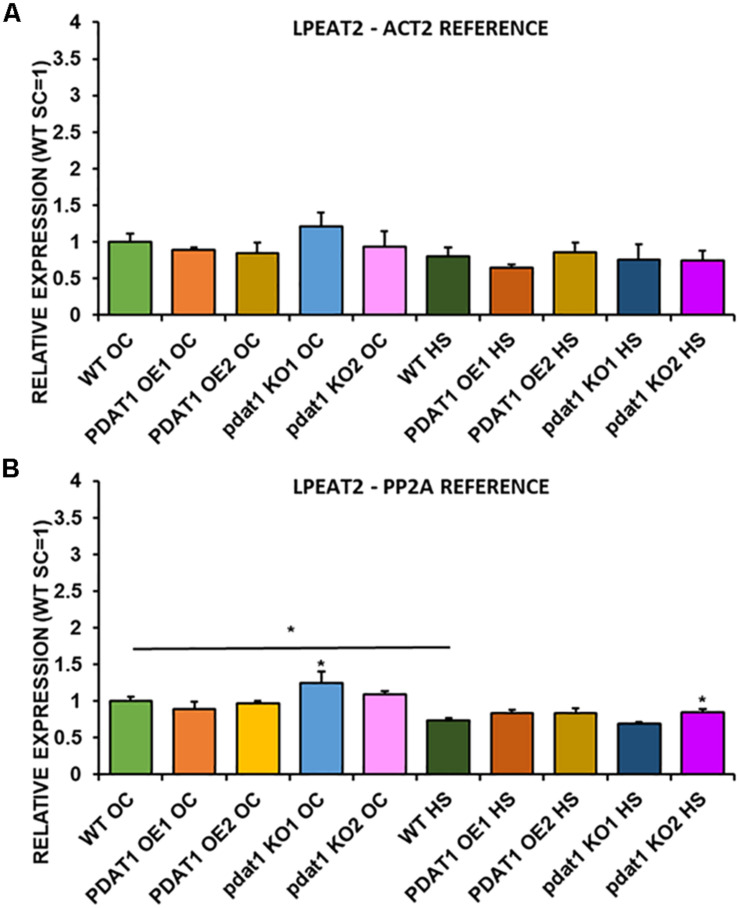
Relative expression of *LPEAT2* in 3-week old rosettes of *Arabidopsis thaliana* in comparison to *ACT2* housekeeping gene **(A)** and *PP2A* housekeeping gene **(B)** as determined by RT-qPCR. WT indicates wild-type line (control), PDAT1 OE1 indicates *AtPDAT1*- overexpressing line 1, PDAT1 OE2 indicates *AtPDAT1*- overexpressing line 2, pdat1 KO1 indicates line 1 with *pdat1* knockout and pdat1 KO2 indicates line 2 with *pdat1* knockout. OC refers to seedlings grown only in optimal conditions, whereas HS refers to seedlings, which were subjected to 2 h heat stress. Error bars indicate standard deviations (SD) between biological replicates (*n* = 3). Single (*) asterisks above error bars indicate significant difference at *p* < 0.05 in a mean difference two-sided test between the tested line and wild-type line grown in the same conditions (OC or HS). Single (*) asterisks above horizontal lines indicate significant difference in a two-tailed Student’s *t*-test at *p* < 0.05 between means of the results two columns at the border of the horizontal line represent.

We first measured relative expression of *LPCAT1* in both optimal and heat-shock conditions throughout all lines investigated in this study (Figure 10). The expression of this gene did not change in any lines in non-stressed plants except for it being lower than control in *PDAT1*-overexpressing line 1 with *PP2A* as a reference. In stress conditions, a significant difference was found between *PDAT1*-overexpressing line 1 and WT with one of the reference genes (*ACT2*). Those differences, however, were not much lower than in WT and they were not found to be significant with the other reference gene nor were they confirmed by expression measured in *PDAT1*-overexpressing line 2. The second *LPCAT* isoenzyme’s expression turned out to be much more diverse (Figure 11). In optimal conditions *LPCAT2* expression was more than two times higher than control in both studied *PDAT1*-overexpressing lines, and with both reference genes. It was also significantly lower in *pdat1* mutant line 1 with both *ACT2* and *PP2A*. Expression of *LPCAT2* grew in all the lines in stress conditions, and was still significantly higher in *PDAT1*-overexpressing lines than in wild-type. *LPEAT1* relative expression (Figure 12) showed no difference between the lines in neither plants subjected to non-stress and stress conditions. The enzyme’s expression increased, however, between plants grown in optimal conditions and plants that endured heat-shock treatment, in all the studied lines. Its isoenzyme, *LPEAT2*, did not have increased expression in *PDAT1*-overexpressing lines neither (Figure 13). In stress conditions *LPEAT2* expression was slightly (yet significantly with *PP2A*) lower after heat-shock than in optimal conditions. Non-stressed *pdat1* mutant lines were exhibiting marginal increase in *LPEAT2* expression.

## Discussion

Phospholipid:diacylglycerol acyltransferase (PDAT) still remains somewhat of an enigma within the enzymes involved in plant lipid biosynthesis. One of the reasons we decided to investigate it was to lift the veil on its mystery a bit more, to further our understanding of PDAT’s role in plant metabolism. The enzyme’s main function was naturally assumed to be connected with its enzymatic activity – production of TAG. However, hitherto reports on *PDAT* overexpression in Arabidopsis lead to a certain ambiguity. There are publications suggesting that *PDAT* overexpression in Arabidopsis translates to higher TAG content (Fan et al., 2013a,b). Transient expression of *PDAT* from *C. sativa* has also led to increase in TAG production in tobacco leaves (Yuan et al., 2017a). However, there are also publications demonstrating lack of TAG production increase in Arabidopsis’ seeds with *PDAT* overexpression (Ståhl et al., 2004; Banaś et al., 2014). In this study, we have found that TAG content in leaves increased in all of the studied lines after heat-shock. Increased TAG accumulation in the stressed plant leave tissue was shown before (Yang and Benning, 2018). However, there were no outright tendencies as to TAG accumulation and *PDAT1* overexpression and knockout. In stressed *pdat1* mutant lines DGAT enzymes may overcompensate for lack of PDAT1 activity, which would be in agreement with previous findings (Zhang et al., 2009).

**FIGURE 14:**
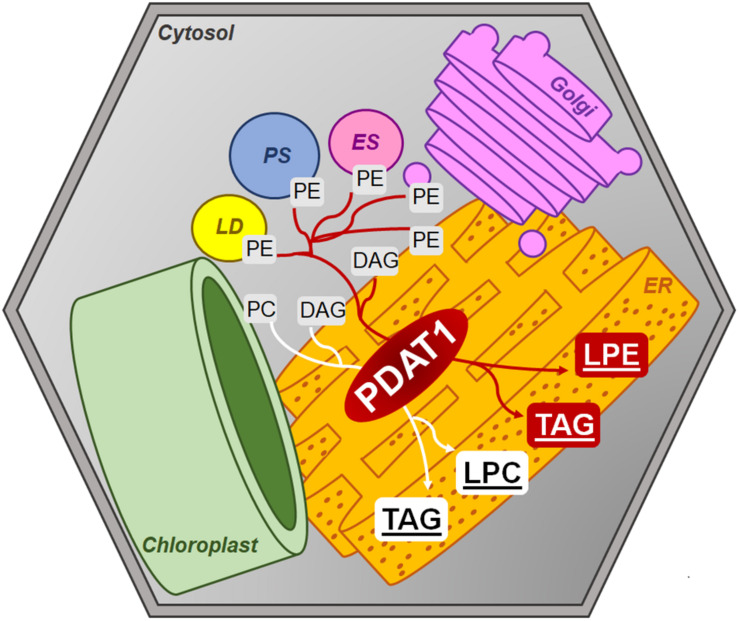
Postulated PDAT1 enzymatic action within plant cells’ cytosolic membranes. DAG, diacylglycerol; ER, endoplasmic reticulum; ES, endosome; Golgi, Golgi apparatus; LD, lipid droplet; LPC, lysophosphatidylcholine; LPE, lysophosphatidylethanolamine; PC, phosphatidylcholine; PE, phosphatidylethanolamine; PS, peroxisome; TAG, triacylglycerol.

Amongst the mixed reports concerning *PDAT* overexpression affecting TAG content, it was shown that even when *PDAT* overexpression in Arabidopsis did not result in elevated TAG levels, it produced plants with increased growth rate and weight in comparison to wild-type (Banaś et al., 2014). Progeny of those *PDAT1* overexpression plants were used in our study and resulted in plants, which were visibly bigger than wild-type control. What is more, T-DNA insert *pdat1* mutants genotyped and selected by us, which did not express the studied gene, were smaller than wild-type. This phenomenon resulted in heightened rosette and root fresh weights of *PDAT1*-overexpressing line and lower equivalent weights of *pdat1* mutant organs, in comparison to control. The level of *PDAT1* expression in a particular Arabidopsis line correlated with the plants aging rate with *PDAT1* overexpression resulting in a plant lifecycle with delayed senescence, whereas *pdat1* knockout aged faster. One possible explanation of *PDAT1*’s role in aging process may be connected to lysophosphatidylethanolamines (LPE). PDAT as a TAG-producing enzyme preferentially utilizes PC and PE as substrates (Figure 14). When PDAT transfers an acyl group from the above-mentioned phospholipids to DAG, what is left as by-products are lysophospholipids: lysophosphatidylcholine (LPC) and LPE. LPE have been long known as plant senescence retardants (Farag and Palta, 1993). Addition of exogenous LPE induces expression of phenylalanine ammonia lyase and acid invertase, pathogenesis-related proteins, which in turn results in anabolic lignin formation and its deposition to cell walls (Cowan, 2009; Hong et al., 2009). *PDAT1* overexpression introduced in Arabidopsis might have resulted in an increase of LPE amount in those plants, which caused its elicitor action, leading to the observed delayed senescence. The opposite might have been true for *pdat1* mutant, which might have had lower LPE amount than wild-type cultivated in the same environmental conditions. Different LPE amounts in different lines may have also contributed to the changes observed in the fresh weight measurements of 4-week-old plants *in vitro*.

One of the proposed possible roles of PDAT in plant metabolism concerns the enzyme being an acyl group donor utilizing membrane phospholipids (PC and PE). PDAT would thus be responsible for regulating the fatty acid composition of cytosolic membrane lipids – which is linked with plant reaction to stress factors (Dahlqvist et al., 2000; Banaś et al., 2014). PDAT’s connection with plant stress response is what we have explored in our research. When we subjected our tested lines to short-term heat exposure followed by a period of 1-week recovery, we noticed a growth boost in *PDAT1*-overexpressing line and wild-type control in comparison to plants of similar age, which were not exposed to the heat shock. This may be due to activation of the stress response mechanisms of plants, which were set off by the 2-h submission to adverse temperature. Knockout lines of *pdat1* in our study exhibited increase in root mass after stress, but not much difference in rosette weight in comparison to control in particular temperature. In previous reports concerning *pdat1* knockout lines response to heat stress, heat has been found to impede *pdat1* knockout mutants in Arabidopsis from accumulating TAG after stress (Mueller et al., 2017). We also show that short exposure to high temperature increases *PDAT1* expression in wild-type plants by at least a 3-fold. It was shown that CaMV 35S promoter-driven expression of a transgene can increase in *Nicotiana benthamiana* leaves after exposing them to short-term 50°C stress, but the increase went down after several hours and constituted at most 1.33 of the relative expression in non-stressed leaves (Boyko et al., 2010). In our study *PDAT1* expression also increases in *PDAT1*-overexpressing lines after heat-treatment, despite the already present *PDAT1* mRNA abundance due to overexpression. This may partly correlate to further weight increase of *PDAT1*-overexpressing lines after high temperature stress. By far the biggest difference in fresh weight between the studied Arabidopsis lines was observed, when 2-week-old seedlings were transferred to a cold chamber and grown there for two additional weeks. Both the wild-type and the *pdat1* mutant did not endure the harmful conditions well, being much smaller than plants grown in optimal conditions and losing pigment, which would foil their ability to bounce back, if transferred back to 23°C. Still, *pdat1* mutants’ leaves and roots weighed less than the wild-type, which means they were even less tolerant to the cold than the control. The *PDAT1*-overexpressing lines were much less sensitive to the cold, with their rosettes weighing four times as much and roots weighing five to seven times as much as the wild-type. Most of the *PDAT1*-overexpressing plants retained the green color, an indication of their kept ability to photosynthesize carbohydrates, which would possibly enable them to recover, if they were placed back in optimal conditions. The importance of *PDAT1* in cold stress has been previously reported in *Camelina sativa* (Yuan et al., 2017a). Low temperatures induced increased *PDAT1* expression by 6-fold (Yuan et al., 2017b), with particular isoforms expression, *CsPDAT1*-A and *CsPDAT1*-C, increasing by 3.5-fold and 2.5-fold, respectively (Yuan et al., 2017a). We have to remember, however, that our experiments were conducted *in vitro* and may not reflect how well *PDAT1* overexpression would facilitate better plant stress response in the natural environment. Further studies have to be performed to resolve these doubts.

As was mentioned above, LPE and LPC are the by-products of PDAT activity. Both are utilized by LPEAT and LPCAT enzymes to synthesize phospholipids. Thus, we had suspected, that in plants with *PDAT1* overexpression we would observe higher activity of LPEAT and LPCAT (being expressed more because of additional substrates available for their enzymatic action). There were no previous reports concerning this topic. Our research shows that indeed *PDAT1* overexpression in Arabidopsis leads to an increase in LPEAT and LPCAT activity in both rosette and root microsomal fractions. The increase in LPCAT activity in rosettes in comparison to control was not as profound as in other cases (but still statistically significant in *PDAT1*-overexpressing line 2) in the presented results, but our preliminary studies showed that activity to be 2.5 times higher. The differences may be due to different microsomal preparations used (that being said, microsomal fractions of a particular set of all the lines were prepared at the same time). The heightened microsomal LPCAT activity in *PDAT1*-overexpressing lines corresponds well with the double increase in *AtLPCAT2* relative expression calculated for the same lines. We also measured *LPEAT* expression *in vitro* and discovered that the relative expression of both *LPEAT* isoenzymes in the investigated *PDAT1*-overexpressing lines did not differ much from the control, which does not confirm the observed increase in microsomal LPEAT activity. This may be due to the different methods of cultivating plants for microsomal preparations and difference in the plants’ age.

It was suggested, that PE-producing LPEAT enzymes may be connected with the regulation of the autophagy intensity (Jasieniecka-Gazarkiewicz et al., 2017). We decided to test, whether *PDAT1* overexpression or knockout would trigger increase or decrease in autophagocytic response, since PE is a major substrate for PDAT. *ATG8a* expression levels, which were measured in all lines subjected and not subjected to heat shock, are considered one of the markers for intensity of plant autophagy. Our findings showed that increase of *ATG8a* relative expression levels after high temperature exposure did not correlate with *PDAT1*-overexpression or *pdat1* knockout in Arabidopsis. These findings may mean that *PDAT1* overexpression’s role in increasing plant fitness after stress does not have to be outright connected with plant autophagocytic response.

The precise reason why *PDAT1* accentuates plant stress response needs to be elucidated further, but its overexpression certainly increases plant’s endurance through disadvantageous environmental conditions. Our research opens possibilities for genetic modification of crops to be more resilient to adverse environment through increased PDAT1 activity. Investigation of PDAT1’s effect on different plant species should continue, in order to assess the benefits of such genetic modifications.

## Data Availability Statement

The original contributions presented in the study are included in the article/Supplementary Material, further inquiries can be directed to the corresponding author/s.

## Author Contributions

KD, KJ-G, and AB conceived and designed the research. AŁ, KD, and SK conducted the experiments. KD and AB analyzed the data. KD wrote the original draft of the manuscript. All authors reviewed and edited the final version.

## Conflict of Interest

The authors declare that the research was conducted in the absence of any commercial or financial relationships that could be construed as a potential conflict of interest.
