# Supplementary material for: Phospholipid:Diacylglycerol Acyltransferase1 Overexpression Delays Senescence and Enhances Post-heat and Cold Exposure Fitness
Source: Front Plant Sci. 2020 Dec 14;11:611897. doi: 10.3389/fpls.2020.611897 (PMC7767865; doi:10.3389/fpls.2020.611897)
Supplement: Supplementary file 1 [file Data_Sheet_1.PDF]

Supplementary Table 1. List of primers used to determine the zygosity of plants of SALK_065334 (*pdat1* KO1) and SALK_032261 (*pdat1* KO2) lines.

Primer Role	Primer Sequence (5′ to 3′)
Left T-DNA Border Primer	ATTTTGCCGATTTCGGAAC
Left Genomic Primer (*pdat1* KO1)	CATGTGGTGTTGCATTTTCAG
Right Genomic Primer (*pdat1* KO1)	TTTTGTTTTCGGTCTTGTTGG
Left Genomic Primer (*pdat1* KO2)	GGTCTACACTGCTGGTGAAGC
Right Genomic Primer (*pdat1* KO2)	ACCCACAAATTGATGCTTTTG

Supplementary Table 2. List of primers used in this study to measure relative gene expression.

Gene of Interest Name	Primer Orientation	Primer Sequence (5′ to 3′)
ACT2	forward	TGGAATCCACGAGACAACCTA
	reverse	TTCTGTGAACGATTCCTGGAC
PP2A	forward	CAGATGTGCTAAAGACGGAGC
	reverse	TGTGCAACACAGTCCTGGG
PDAT1	forward	TGGCTGCTGACTACTTTGCTC
	reverse	ACCTCTGTGTTCTGAAACGAAAG
LPEAT1	forward	TATGGGACGATGGGTTTGGGTCCTT
	reverse	ACGATAAGGAGCAGAAAACAGC
LPEAT2	forward	AGGGATGAAAGCACCGAGTTG
	reverse	ACATCAAGGAAGAATGCTGTGC
ATG8a	forward	ATGATCTTTGCTTGCTTGAAATTC
	reverse	AGCCTTCTCCACAATCACG
LPCAT1	forward	ACCTCTCCTTTGGCTTCTCC
	reverse	TCCTCCTTCTTTCCACGCAT
LPCAT2	forward	AGGAAAGAGACCATCGCCTT
	reverse	CGTGAAACCCGCCATGTATT

Supplementary Table 3. Differences in occurrence of key *Arabidopsis thaliana* life cycle events between wild-type (WT), PDAT1-overexpressing lines (PDAT1 OE1 and OE2) and pdat1 knock-out lines (pdat1 KO1 and KO2). Results are presented as a day after sowing in which the event of interest occurred followed by standard deviation (after ±). Asterisks indicate significant difference in comparison to control (WT) in a mean difference two-sided test (p<0.0001, n=6).

**Life cycle event**	**WT**	***PDAT1* OE2**	***PDAT1* OE2**	***pdat1* KO1**	***pdat1* KO2**
First flower buds (0 DAF)	27 ± 0.52	23* ± 1.21	23* ± 1.05	27 ± 0.63	27 ± 0.75
First flower open	32 ± 0.75	28* ± 0.52	27* ± 0.41	34 ± 0.82	35 ± 0.55
First Yellow Leaf	43 ± 1.47	48 ± 1.64	49 ± 1.87	40 ± 1.17	40 ± 0.82
All-Yellow Rosette	54 ± 0.55	60* ± 2.13	62* ± 2.8	52 ± 1.38	51 ± 1.05

Cessation of Flowering	60 ± 1.37	60 ± 1.51	58 ± 1.72	61 ± 0.63	61 ± 1.17

Supplementary Table 4. Comparison of endogenous posphatidylcholine (PC) and microsomal protein concentration between isolated microsomal fractions used for conducting presented *in vitro* assays.

Arabidopsis line	Roots' Microsomal Fraction	Rosettes' Microsomal Fraction
**WT**	12.45 µg/nmol PC	10.06 µg/nmol PC
**PDAT1 OE1**	10.60 µg/nmol PC	8.15 µg/nmol PC
**PDAT1 OE2**	8.93 µg/nmol PC	8.24 µg/nmol PC

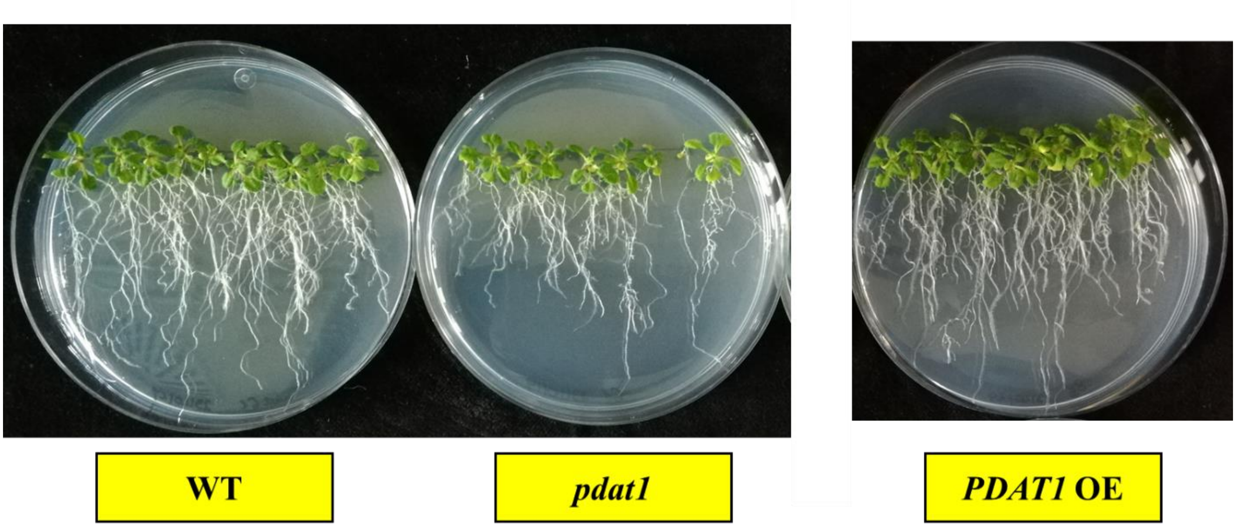

Supplementary Figure 1. Cultivation of three-week-old *Arabidopsis thaliana* lines *in vitro* in optimal conditions: wild-type control (WT), *pdat1* knock-out mutant KO2, *PDAT1* OE – *PDAT1*-overexpressing line OE2.

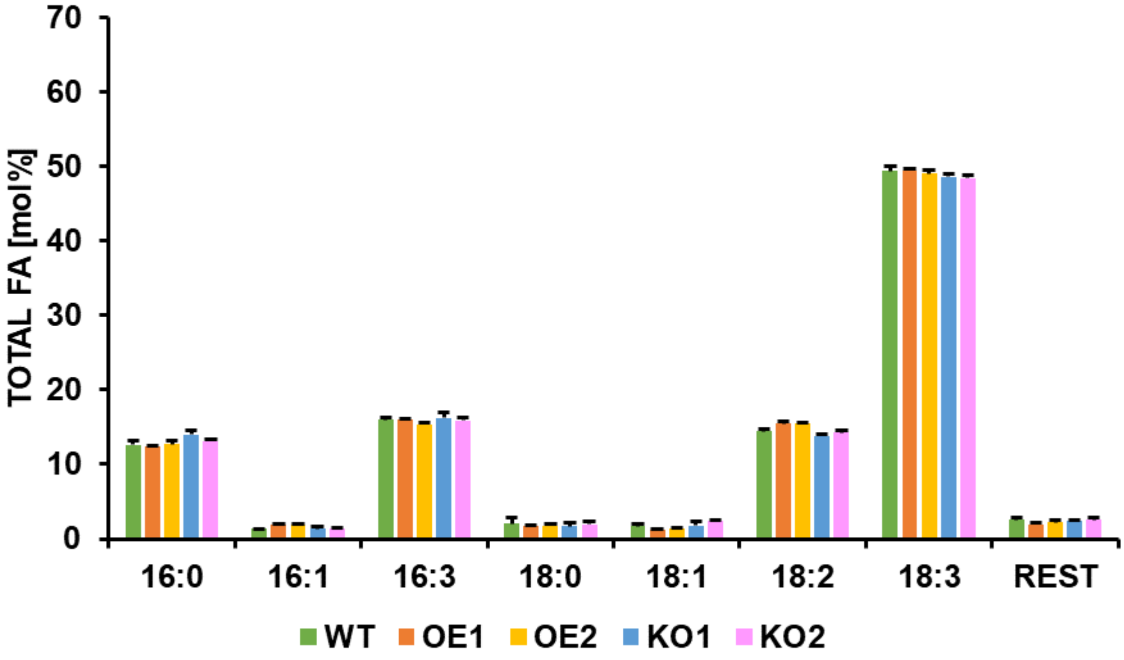

Supplementary Figure 2. Comparison of total fatty acid (FA) content in four-week-old Arabidopsis rosettes grown in optimal conditions. Comparison was made between the following five lines: wild-type control (WT), two *PDAT1*-overexpressing lines OE1 and OE2 and two *pdat1* knock-out mutants KO1 and KO2.

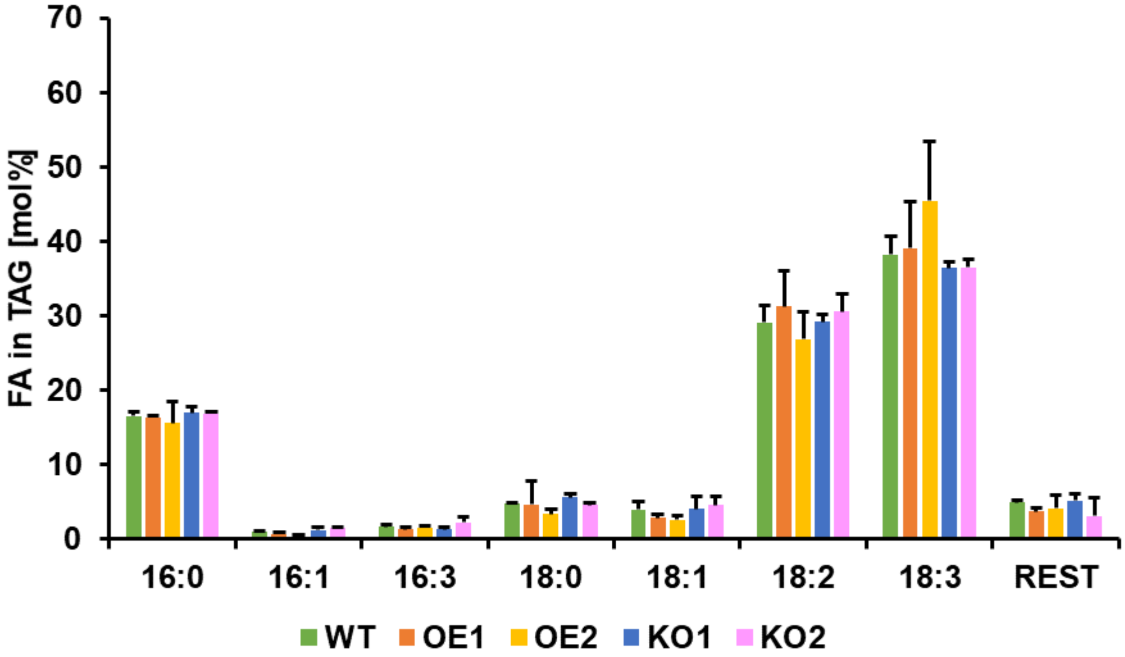

Supplementary Figure 3. Comparison of fatty acid (FA) composition in triacylglycerol (TAG) lipid fraction of four-week-old Arabidopsis rosettes grown in optimal conditions. Comparison was made between the following five lines: wild-type control (WT), two *PDAT1*-overexpressing lines OE1 and OE2 and two *pdat1* knock-out mutants KO1 and KO2.

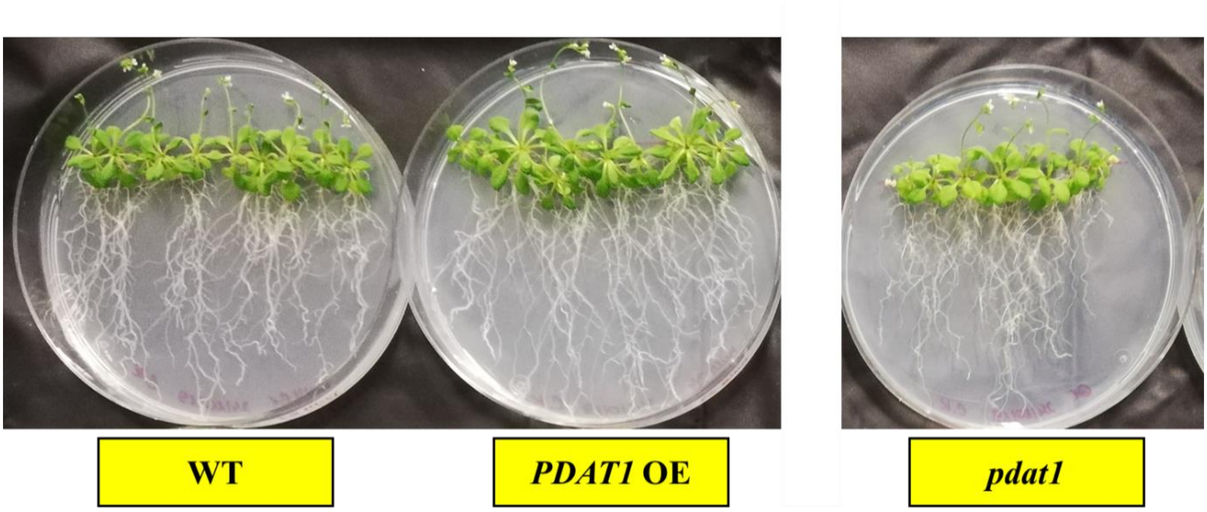

Supplementary Figure 4. Four-week-old *Arabidopsis thaliana* lines *in vitro*: wild-type control (WT), *pdat1* knock-out mutant KO2, *PDAT1* OE – *PDAT1*-overexpressing line OE2. All plants were grown in optimal conditions for three weeks, then exposed to two-hour 40 °C heat-shock and then put in optimal conditions for additional week to recover.

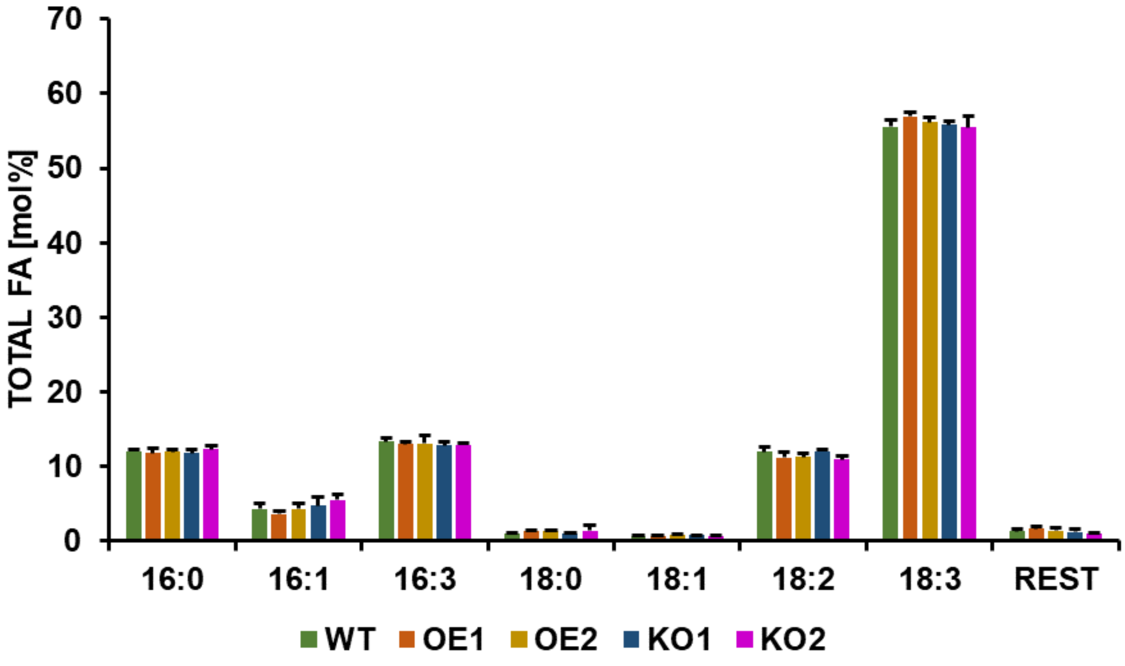

Supplementary Figure 5. Comparison of total fatty acid (FA) content in four-week-old Arabidopsis rosettes, which were subjected to two-hour heat-shock (40 °C) at three weeks old. Comparison was made between the following five lines: wild-type control (WT), two *PDAT1*-overexpressing lines OE1 and OE2 and two *pdat1* knock-out mutants KO1 and KO2.

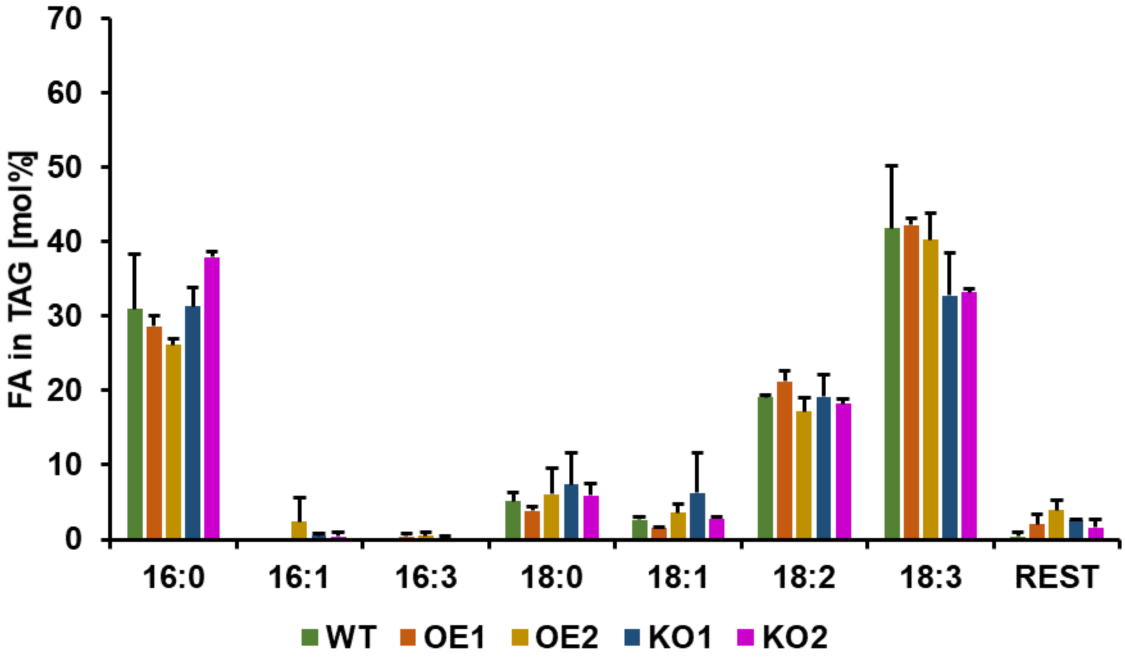

Supplementary Figure 6. Comparison of fatty acid (FA) composition in triacylglycerol (TAG) lipid fraction of four-week-old Arabidopsis rosettes, which were subjected to two-hour heat-shock (40 °C) at three weeks old. Comparison was made between the following five lines: wild-type control (WT), two *PDAT1*-overexpressing lines OE1 and OE2 and two *pdat1* knock-out mutants KO1 and KO2.

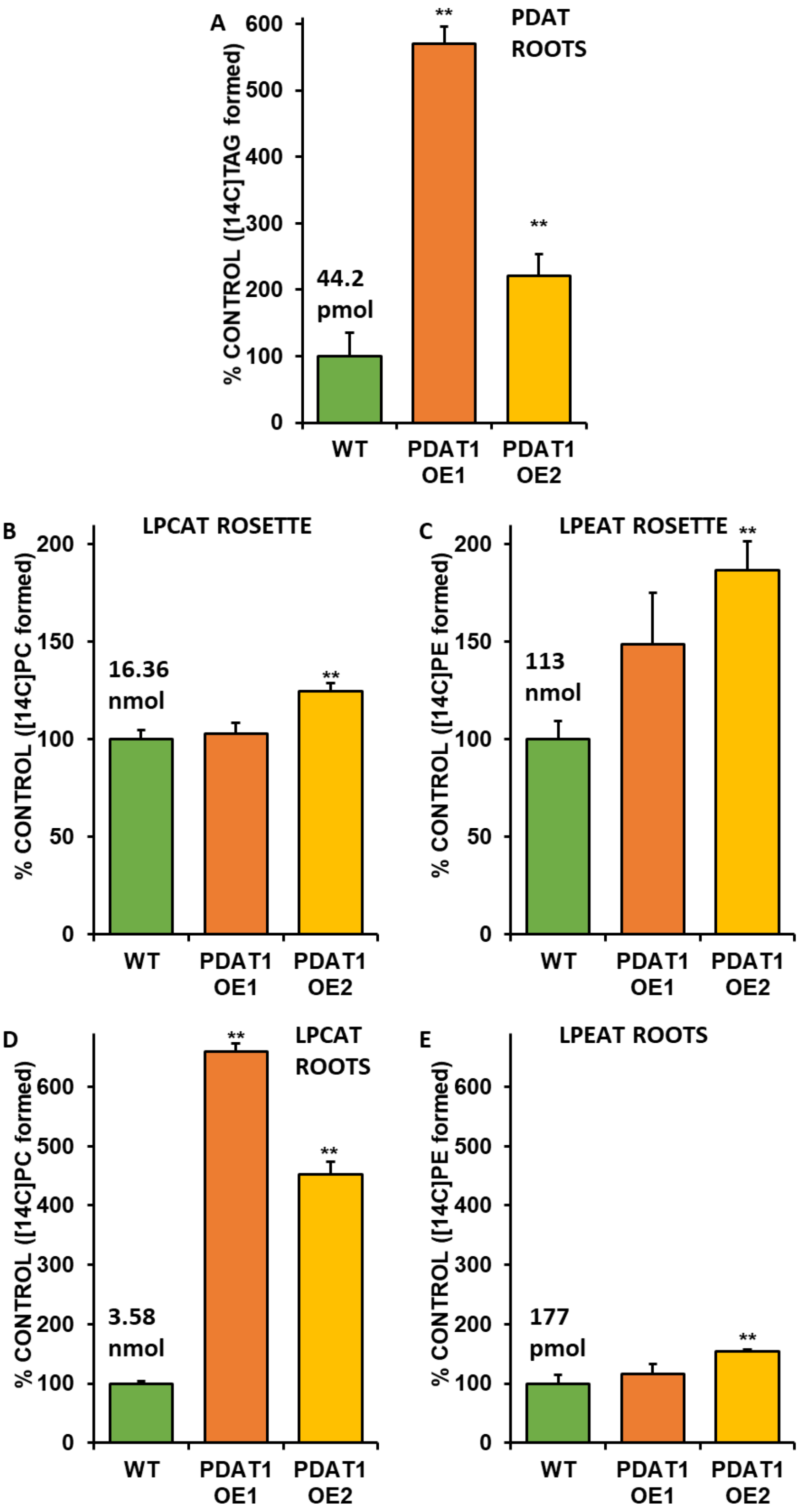

Supplementary Figure 7. Endogenous enzyme activity measured in vitro in root **(A, B, C)** and rosette **(D, E)** microsomal fractions from Arabidopsis wild-type control (WT) and PDAT1-overexpressing lines (PDAT1 OE1 and PDAT1 OE2). Comparison of enzyme activity of PDAT **(A)**, LPCAT **(B, D)** and LPEAT**(C, E)** between the two lines. Values above

the WT bars correspond to 100% activity for each chart measured as pmol or nmol of [14C] enzymatic activity product (TAG for PDAT, PC for LPCAT and PE for LPEAT) synthesised during 1h reaction, utilising microsomal fraction equivalent to 1 nmol of endogenous PC. Error bars indicate standard deviations (SD) between biological replicates (n=3). Single (*) or double (**) asterisks indicate significant difference between means in comparison to control (WT) in a two-tailed Student's t-test at p<0.05 or at p<0.01, respectively.

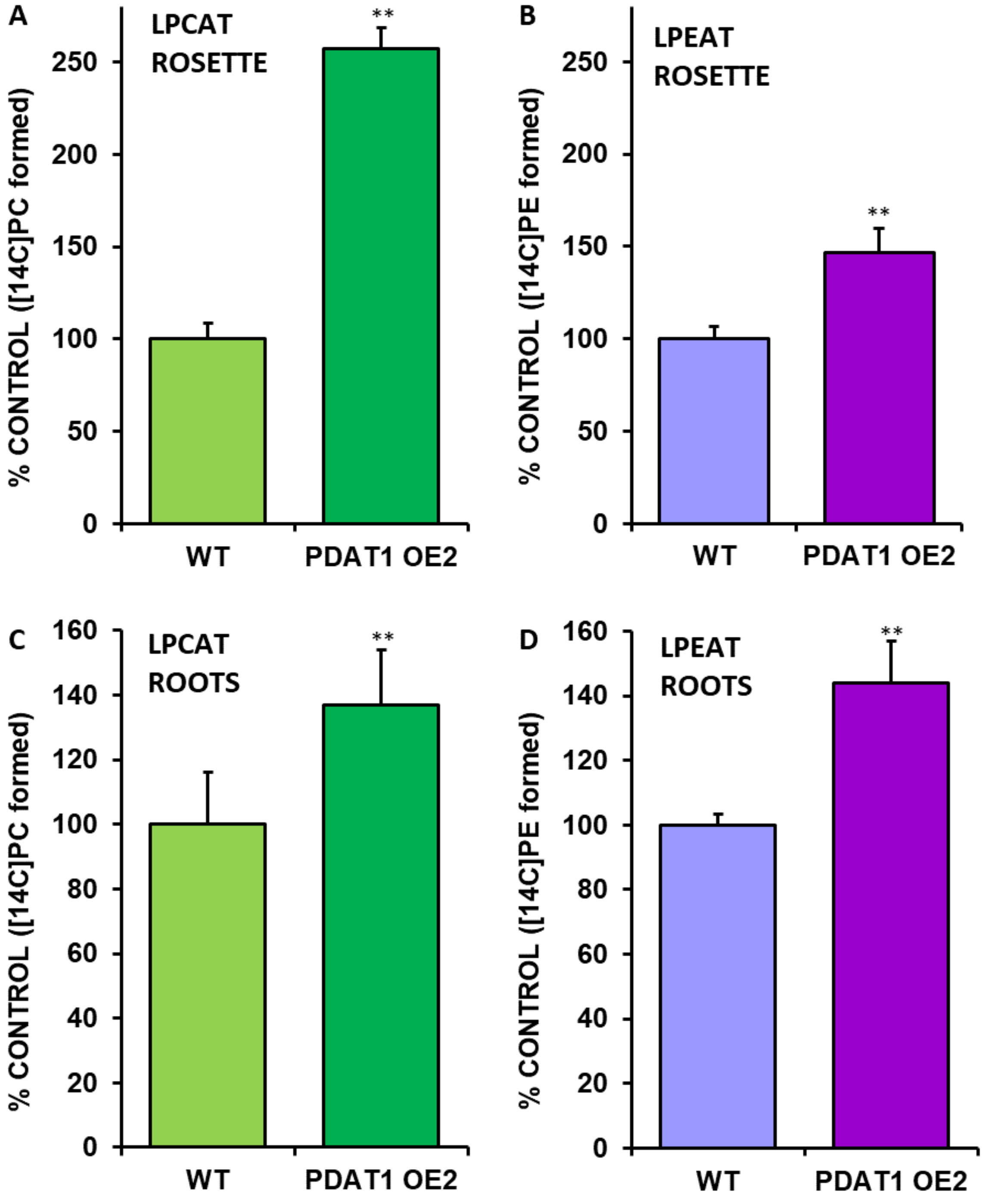

Supplementary Figure 8. Preliminary studies in endogenous enzyme activity measured *in vitro* in rosette **(A, B)** and root **(C,D)** from different microsomal fractions from Arabidopsis wild-type control (WT) and *PDAT1*-overexpressing line 2 (*PDAT1* OE2), than those presented in the main article. Comparison of enzyme activity of LPCAT **(A, C)** and LPEAT

(B, D) between the two lines. Error bars indicate standard deviations (SD) between technical replicates (n=3). Double asterisks (**) indicate significant difference in comparison to control (WT) in a mean difference two-sided test at $p<0.01$.
